# SHF Acts as a Novel Tumor Suppressor in Glioblastoma Multiforme by Disrupting STAT3 Dimerization

**DOI:** 10.1002/advs.202200169

**Published:** 2022-07-17

**Authors:** Jingjing Wang, Zixuan Huang, Li Ji, Cheng Chen, Quan Wan, Yu Xin, Zhening Pu, Koukou Li, Jiantong Jiao, Ying Yin, Yaling Hu, Lingli Gong, Rui Zhang, Xusheng Yang, Xiangming Fang, Mei Wang, Bo Zhang, Junfei Shao, Jian Zou

**Affiliations:** ^1^ Department of Laboratory Medicine Wuxi People's Hospital of Nanjing Medical University Wuxi Jiangsu 214023 China; ^2^ Center of Clinical Research Wuxi People's Hospital of Nanjing Medical University Wuxi Jiangsu 214023 China; ^3^ Department of Neurosurgery The Affiliated Wuxi Second Hospital of Nanjing Medical University Wuxi Jiangsu 214002 P. R. China; ^4^ Key Laboratory of Industry Biotechnology School of Biotechnology Jiangnan University Wuxi Jiangsu 214122 P. R. China; ^5^ Department of Neurosurgery The Affiliated Wuxi People's Hospital of Nanjing Medical University Wuxi Jiangsu 214023 China; ^6^ Department of Radiology Wuxi People's Hospital of Nanjing Medical University Wuxi Jiangsu 214023 China

**Keywords:** dimerization, DNA methylation, DNA methyltransferase 1, glioblastoma, SH2 domain‐containing adapter protein F, signal transducer and activator of transcription 3

## Abstract

Sustained activation of signal transducer and activator of transcription 3 (STAT3) is a critical contributor in tumorigenesis and chemoresistance, thus making it an attractive cancer therapeutic target. Here, SH2 domain‐containing adapter protein F (SHF) is identified as a tumor suppressor in glioblastoma Multiforme (GBM) and its negative regulation of STAT3 activity is characterized. Mechanically, SHF selectively binds and inhibits acetylated STAT3 dimerization without affecting STAT3 phosphorylation or acetylation. Additionally, by blocking STAT3‐DNMT1 (DNA Methyltransferase 1) interaction, SHF relieves methylation of tumor suppressor genes. The SH2 domain is documented to be essential for SHF's actions on STAT3, and almost entirely replaces the functions of SHF on STAT3 independently. Moreover, the peptide C16 a peptide derived from the STAT3‐binding sites of SHF inhibits STAT3 dimerization and STAT3/DNMT1 interaction, and achieves remarkable growth inhibition in GBM cells in vitro and in vivo. These findings strongly identify targeting of the SHF/STAT3 interaction as a promising strategy for developing an optimal STAT3 inhibitor and provide early evidence of the potential clinical efficacy of STAT3 inhibitors such as C16 in GBM.

## Graphical abstract

SH2 domain‐containing adapter protein F (SHF) is identified as tumor suppressor in glioblastoma and its negative regulation of STAT3 activity is characterized by disrupting acetylated STAT3 dimerization and STAT3‐DNMT1 interaction, thereby relieving methylation of tumor suppressor genes, and a peptide targeting STAT3‐binding sites of SHF is demonstrated as a suppressive strategy.

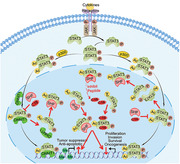

## 1 Introduction

Glioblastoma Multiforme (GBM) is the most common malignant primary brain tumor in adults, with a rapid growth rate and high local recurrence.^[^ ^1^ ^]^ The poor prognosis of GBM is due to therapeutic resistance and recurrence, in addition to complex molecular changes which remain a great challenge for translational researchers. Signal transducer and activator of transcription 3 (STAT3) is a critical mediator of the occurrence, development, and prognosis of GBM. Sustained activation of STAT3 frequently occurs in GBM and has been recognized as a potential treatment target.^[^ ^2^ ^]^

Typically, STAT3 is activated through phosphorylation induced by cytokine receptor associated kinases such as Janus kinases (JAKs) and receptor tyrosine kinases following ligand binding.^[^ ^3^ ^]^ Upon phosphorylation, a STAT3 dimer is formed and translocated to the nucleus, binding to specific DNA‐response elements in the promoter of target genes and inducing or suppressing gene expression. A critical step of STAT3 activation is the dimerization of two STAT3 monomers. Thus, the inhibition of STAT3 dimerization presents an attractive target for the abolishment of STAT3 biological functions.^[^ ^4^, ^5^, ^6^ ^]^ STAT3‐STAT3 dimerization relies on the reciprocal interaction of its SH2 domains. Although disrupting the reciprocal interaction of the SH2 domains has demonstrated efficacy for the inhibition of STAT3 activity, it remains a challenging target for drug discovery, which restricts clinical development.^[^ ^7^, ^8^, ^9^, ^10^, ^11^, ^12^ ^]^

The SH2 domain is a structurally conserved protein domain contained in Src and many other intracellular signal‐transducing proteins.^[^ ^13^ ^]^ Proteins containing the SH2 domain can dock with phosphorylated tyrosine residues on other proteins. Among the SH2 containing proteins, the SH2 domain‐containing adapter proteins comprise a subfamily which plays multifunctional roles across a range of cellular activity. These proteins operate by linking activated tyrosine kinase receptors to downstream signaling components.^[^ ^14^ ^]^ A highly conserved and common feature of these proteins is that the SH2 domain is located at the C‐terminal. SH2 domain‐containing adapter protein F (SHF) has a unique protein structure compared to other family members, namely in that its N‐terminal lacks a proline‐rich region and phosphotyrosine‐binding (PTB) domain.^[^ ^15^, ^16^ ^]^ This makes SHF the only family member negative regulatory action on tyrosine kinase receptor‐oriented signal pathways.^[^ ^16^, ^17^, ^18^ ^]^ Although there are only a few studies on SHF, there are clues that suggest the negative association of SHF with STAT3.^[^ ^16^ ^]^

In the present study, we observed that SHF is the most significant downregulated gene among SH2 domain‐containing adapter proteins in GBM. Its downregulation predicts poor patient survival and is implicated in high carcinogenicity. Functional experiments identified SHF as a tumor suppressor in GBM. Mechanical investigation further disclosed that SHF impairs GBM tumorigenesis by binding to and inhibiting STAT3 dimerization through its SH2 domain. Additionally, by blocking STAT3‐DNMT1 interaction, SHF relieves methylation of multiple tumor suppressor genes. Using protein‐protein docking, a peptide was derived from the STAT3‐binding sites of SHF and demonstrated to be efficacy as a GBM suppressive strategy.

## 2 Results

### 2.1 Downregulation of SHF in GBM Predicts Poor Outcomes

**Figure 1:**
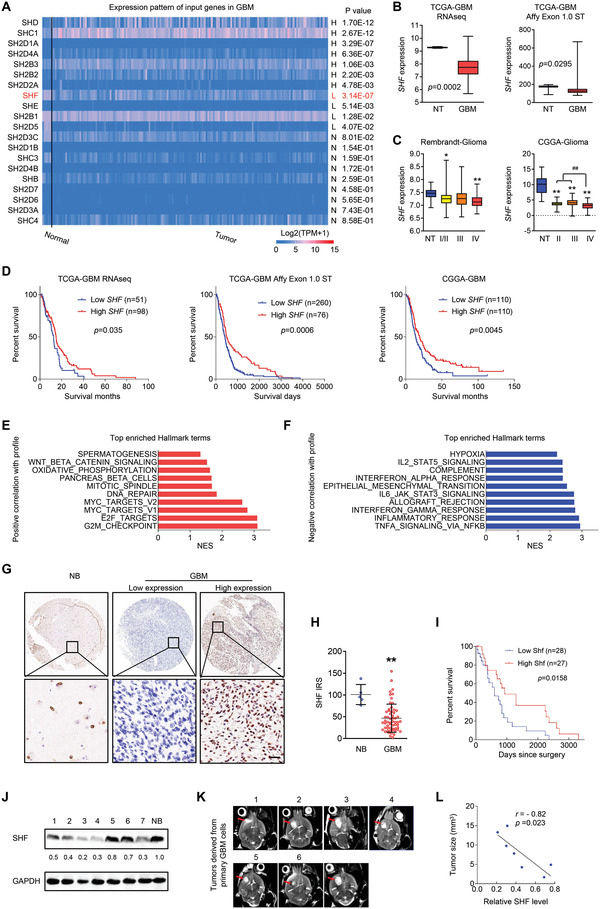
SHF downregulation is associated with tumorigenesis and predicts poor patient prognosis in GBM. A) Comparison of transcript levels of genes coding the SH2 domain‐containing protein between normal and GBM brains according to the TCGA‐GBM dataset (http://ualcan.path.uab.edu). B) Statistical analysis of SHF mRNA expression between non‐tumor brain tissues (NT) and GBMs in TCGA‐GBM datasets (Student's *t*‐test). C) Statistical analysis of *SHF* mRNA expression across different grades of gliomas derived from the indicated datasets (*p < 0.05, **p < 0.01, two‐tailed Student's *t*‐test). D) Overall survival analysis based on SHF mRNA expression in the indicated GBM datasets (Kaplan–Meier survival test). E–F) The top enriched hallmark gene categories identified by GenePattern. NES value of 1.0 and NOM *p* < 0.05 were used as visual thresholds. G) Immunohistochemical (IHC) analysis of SHF protein expression in a tissue array containing normal brains (NB) and primary GBM tissues. Bars, 50 µm. H) SHF protein was frequently decreased in GBM compared with normal brains (**p < 0.01, Student's *t*‐test). I) Overall survival analysis based on SHF expression in GBM tissues. Groups were ranked according to SHF IHC scores. The median expression of SHF was used as a cutoff (Log‐rank *χ*2 = 5.823, *P* = 0.0158). J) Western blot assay of SHF expression in 3D‐cultured cells derived from primary GBM tissues. GAPDH served as a loading control. The relative expression of SHF is listed. K) Representative MRI images of intracranial xenografts derived from 3D‐cultured cells. Red arrows indicate tumors. L) The correlation analysis between SHF expression and tumor size. (*p*‐ and *r*‐value indicated, Spearman's correlation analysis).

An overview of genes coding SH2 domain‐containing adapter protein across normal and GBMs brains was performed via the TCGA‐GBM dataset derived from UALCAN (http://ualcan.path.uab.edu/). SHF was found to be the most significantly downregulated gene (**Figure** **1**A). The SHF transcriptional expression was further characterized across different TCGA‐GBM datasets (Figure 1B). Additional analysis of the glioma dataset derived from Rembrandt and CGGA datasets showed that SHF mRNA was downregulated both in low and high grade gliomas, compared with non‐tumor brain tissues (Figure 1C). The lowest SHF mRNA was observed in grade IV, compared with low grade gliomas (*p* < 0.01) (Figure 1C). Survival analysis predicted that GBM patients with higher SHF expression would have a favorable survival outcome compared with those with a lower SHF expression (Figure 1D; log‐rank survival analysis). To explore the potential mechanism of SHF regulation in GBM, a gene set enrichment analysis (GSEA) was performed based on genes correlated with SHF derived from TCGA‐GBM RNAseq dataset. The results of the top enriched Hallmark terms illustrated that genes positively correlated with SHF were enriched in cell cycle regulation, Myc regulation and DNA repair processes (Figure 1E). Genes negatively correlated with SHF were associated with cytokine response and related signaling pathways, epithelial mesenchymal transition (EMT), and hypoxia (Figure 1F). To determine the significance of SHF in human GBM, immunohistochemistry (IHC) was performed using a GBM tissue microarray containing 58 human tumor samples and 5 normal human brain samples. As shown in Figure 1G, SHF was primarily localized in the nuclei of normal brain and GBM cells. A quantitative analysis further indicated that SHF expression in GBMs were substantially lower than in normal brains (Figure 1H). For survival analysis, the median expression level of SHF in GBM was used as a cutoff. Of the 55 GBM tissues with detailed survival information, patients with higher SHF were expected to have a better clinical prognosis than those with lower SHF (Figure 1I), regardless of age and sex (Table S1, Supporting Information and Supplementary Data 4). To further clarify the contribution of SHF to GBM pathology, we established a patient‐derived xenograft model. Since the primary GBM tissue could not achieve quantity consistency and reach the standard of transplantation, a 3D‐culture of mixed primary cells derived from GBM tissue was applied for expansion and cryopreservation in subsequent research. The expression of SHF in 3D‐cultured cells were analyzed by Western blot (Figure 1J). Next, we investigated whether SHF expression was associated with tumorigenicity in orthotopic xenograft mouse models using 3D‐cultured cells from patient‐derived GBMs (Figure 1K). As shown in the correlation analysis, the expression levels of SHF in these cells were negatively associated with the tumorigenic potential (Figure 1L).

### 2.2 SHF is a Tumor Suppressor in GBM

**Figure 2:**
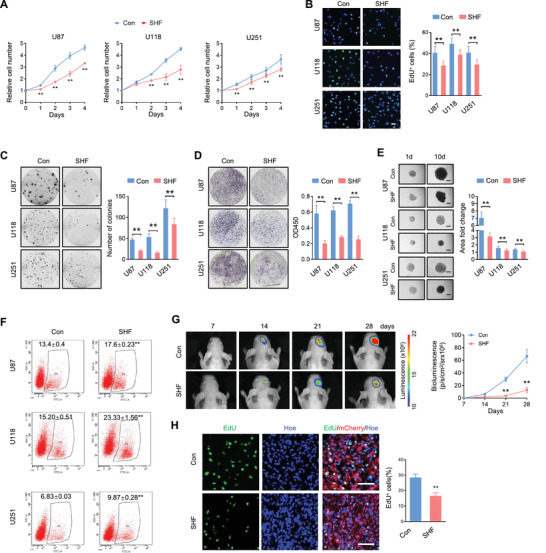
SHF suppresses proliferation, invasion, tumor sphere growth and increases chemosensitivity of GBM cells. A) Proliferation of indicated cells expressing SHF (SHF) or control vectors (Con) as detected by CCK8 assay (*n* = 6, Student's *t*‐test, **p < 0.01). B) EdU‐labeling assay detecting the DNA replication of the indicated cells (*n* = 6, Student's *t*‐test, **p < 0.01). Data was expressed as the percentage of EdU positive cells (Green) to total cells indicated by Hoechst labeling (Blue). C) Colony formation of control cells or ectopic SHF expressing cells (left) and the accompanying statistical analysis (right; *n* = 6, Student's *t*‐test, **p < 0.01). D) Transwell invasion assay of the indicated cells (left) and the statistical analysis (right; *n* = 6, Student's *t*‐test, **p < 0.01). E) 3D‐tumor spheroids growth was recorded (left) and quantitatively analyzed (right; *n* = 12, **p < 0.01, Student's *t*‐test). Bars, 200 µm. F) Annexin V staining flow cytometry assay of cells treated with Dox (2 µm) for 48 h (*n* = 4, Student's *t*‐test, **p < 0.01). G) Bioluminescent images of intracranial xenografts derived from the implantation of the indicated cells (left) and tumor volume statistical analysis (right; *n* = 7, Student's *t*‐test, **p < 0.01). H) Representative immunofluorescence (IF) images of xenograft tumor sections with EdU and mCherry (left) and the statistic of the fraction of EdU‐positive cells (right). Positive cells were quantified 20 randomly selected fields per mouse (*n* = 7, Student's *t*‐test, **p < 0.01). Scale bars, 50 µm.

Next, the functions of SHF were investigated in GBM cell lines. Since SHF is expressed relatively lower in GBM cell lines (Figure S1A, Supporting Information), we established GBM cell lines with ectopic expression of SHF for following studies (Figure S1B, Supporting Information). Cell growth assay showed that ectopic SHF expression significantly impaired cell growth in the GBM cell lines (**Figure** **2**A). Subsequent functional experiments revealed that cells with ectopic SHF exhibited lower DNA replication (Figure 2B) and clonogenicity (Figure 2C), as well as weaker invasion potential (Figure 2D). Accordingly, 3D‐tumor sphere assay showed that the introduction of ectopic SHF impaired the growth of the tumor spheroids derived from the indicated GBM cells (Figure 2E). For cellular survival, ectopic SHF expression failed to induce cell apoptosis in normal culture conditions (Figure S2A, Supporting Information). This resulted in an enhancement of cellular apoptosis induced by DOX (Figure 2F). The increase of DNA fragmentation (Figure S2B, Supporting Information), cleaved Caspase‐3, and the decrease of anti‐apoptosis protein Bcl‐xL (Figure S2C, Supporting Information) was induced by SHF expression with the stress of DOX, further suggesting that SHF possess the function of promoting chemotherapy sensitivity of GBM cells. To further verify the in vivo tumor inhibition of SHF, an intracranial xenograft tumor growth assay was performed in nude mice using luciferase labeled U87 cells (Figure 2G). It showed that tumors derived from U87 cells with ectopic SHF grew more slowly than those derived from the control cells. EdU incorporation assay further demonstrated that SHF reduced tumor cell DNA replication and proliferation capacity (Figure 2H). These results suggest that SHF acts as a tumor suppressor by inhibiting GBM cell tumorigenicity and promoting drug sensitivity.

### 2.3 SHF Negatively Regulates STAT3 Transcriptional Activity by Inhibiting STAT3 Dimerization

**Figure 3:**
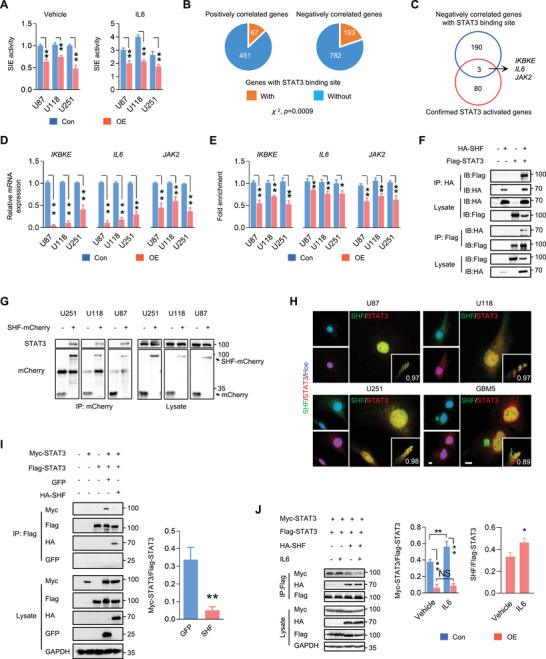
SHF negatively regulates STAT3 activity via binding and inhibition of STAT3 dimerization. A) Luciferase assay of STAT3 transcriptional activity in the indicated cells. IL‐6 (20 ng ml^−1^) was added 6 h before the assay (*n* = 3, Student's *t*‐test, **p < 0.01). B) The pie plots demonstrate the number of SHF‐associated genes positively correlated or negatively correlated with a STAT3 binding site in the promoter (Fisher's exact test). C) The pie plot showing the genes derived from a cross analysis with confirmed STAT3 activated genes and SHF‐associated genes negatively correlated with those demonstrating STAT3 binding evidence in the promoter. D) qRT–PCR assay measuring the expression of previously identified genes in the indicated cells (*n* = 3, Student's *t* test, **p < 0.01). E) The enrichment of STAT3 binding to the promoter of target genes was examined by ChIP‐qPCR (*n* = 3, Student's *t* test, *p < 0.05, **p < 0.01). F) Immunoprecipitation (IP) analysis demonstrating the interaction between SHF and STAT3. 293T cells were transfected with the indicated vectors followed by IP using HA or Flag antibody. The amounts of STAT3 or SHF were detected by immunoblot (IB). The input amounts of SHF and STAT3 were also detected as controls. G) IP analysis showing the interaction between SHF and endogenous STAT3 in GBM cells expressing ectopic SHF‐mCherry. H) Double IF staining of SHF and STAT3 in GBM cells. GBM5 is an established primary GBM cell line. Hoechst (Hoe) stained the nuclei. The vignettes showed the colocalization values of SHF and STAT3. I) IP assay (left) and the statistical analysis (right) indicating that SHF disrupts the dimerization of STAT3 (*n* = 3, Student's *t* test, **p < 0.01). 293T cells were transfected with the indicated vectors followed by IP using Flag antibody. The GFP vector worked as an unrelated control protein. J) IP assay (left) and the accompanying statistical analysis (right) of STAT3 dimerization and STAT3‐SHF interaction with or without IL6 stimulation (*n* = 3, Student's *t* test, *p < 0.05, **p < 0.01). 293T cells were transfected with the indicated vectors and treated with IL6 (10 ng mL^−1^) for 2 h before harvest.

To explore whether STAT3 is mechanically involved in GBM suppression of SHF, the overall STAT3 transcriptional activity was evaluated in GBM cells by a SIE reporter system used previously.^[^ ^19^ ^]^ As shown in **Figure** **3**A, ectopic SHF expression resulted in a significant decrease of STAT3 activity in GBM cells, persisting even under IL6 stimulation. To determine how the inhibition of SHF might impact STAT3 DNA binding ability, STAT3‐binding genes were cross‐referenced with SHF interacting genes and screened (Figure 3B). The SHF‐correlated genes were derived from GlioVis data portal, based on TCGA_GBM RNAseq dataset (Supplementary Data 5). The 3097 genes with STAT3 binding evidence in the promoter were identified from the STAT3‐23295773‐U87‐HUMAN transcription factor binding site profile available from the CHEA Transcription Factor Binding Site Profiles dataset (http://amp.pharm.mssm.edu/Harmonizome/gene_set/STAT3‐23295773‐U87‐HUMAN/CHEA+Transcription+Factor+Binding+Site+Profiles; Supplementary Data 6). Results indicated that of the 518 positively correlated genes, 67 genes had a STAT3 binding site at the promoter (Supplementary Data 7). Of the 975 negatively correlated genes, 193 had a STAT3 binding site at the promoter (Supplementary data 8). Statistical analysis revealed that significantly more SHF‐relevant genes with a STAT3 binding region were negatively correlated than positively correlated, suggesting SHF is a negative regulator of STAT3 transcriptional activity. Next, the 193 negatively correlated genes were cross referenced with other known STAT3 activated genes (Figure 3C). The confirmed STAT3 activated genes were derived from TRRUST database (https://www.grnpedia.org/trrust/result.php?gene=STAT3&species=human&confirm=0) and the genes are listed in Supplementary Data 9. Three established STAT3 target genes were selected and applied to the following analysis. As shown in Figure 3D, SHF overexpression resulted in a significant decrease of these genes in GBM cells. Chromatin immunoprecipitation (ChIP) assay using primers targeting the promoters of the selected genes was used to further explore whether SHF inhibits STAT3 DNA binding capacity. SHF overexpression decreased DNA binding of STAT3 to the targeted genes’ promoters in GBM cells (Figure 3E). Given that dimerization between two STAT3 monomers in the nucleus is a vital process for active STAT3 binding to specific DNA and for regulation of target gene expression, we explored the possibility that the disruption of dimerization may be involved in the inhibitory ability of SHF on STAT3 activity. To this end, BiFC analysis (Figure S3A,B, Supporting Information) was performed to confirm STAT3 forming dimers in GBM cells. The interaction between SHF and STAT3 was demonstrated in 293T cells co‐transfected with SHF and STAT3 plasmids followed by bi‐directional immunoprecipitation (IP) analysis (Figure 3F). Ectopic SHF was further determined to bind endogenous STAT3 (Figure 3G) and colocalize with STAT3 in nuclei of GBM cell lines (Figure 3H). Moreover, the binding affinity between SHF and STAT3 was confirmed by BiFC analysis (Figure S3C, Supporting Information) and FRET assay (Figure S3E, Supporting Information). Subsequently, STAT3 plasmids with different tag were used to monitor the STAT3 dimerization process. The IP analysis showed that two monomers of STAT3 could bind to each other (Figure 3I). Compared to GFP protein, SHF effectively disrupted STAT3 dimerization without affecting STAT3 expression (Figure 3I). The inhibitory effect of SHF on STAT3 dimerization was further defined by FRET assay (Figure S3F, Supporting Information). More importantly, this inhibition worked even in the presence of IL6 (Figure 3J), indicating that inhibitory mechanism of SHF is strong enough to withstand even enhanced STAT3 activation signals induced by IL6. Additionally, these results showed an increased number of SHF‐bound STAT3 in cells stimulated with IL6. STAT3 not only forms homodimers, but also forms heterodimers with STAT1 or STAT5.^[^ ^20^ ^]^ SHF binding of STAT3 allows the possibility that SHF binds with either STAT1 or STAT5. As shown in Figure S4A, Supporting Information, ectopic SHF bound endogenous STAT3 and STAT1, but not STAT5 in U87 and U251 cells, while no detectable interaction between STAT3 and STAT1 or STAT5 was observed in U87 and U251 cells, and SHF introduction did not alter this interaction (Figure S4B, Supporting Information). Given that disruption of STAT3‐dimerizaton is a potential and effective anti‐cancer target,^[^ ^9^, ^11^ ^]^ these results indicate that the tumor suppression action of SHF is mechanically targeted toward STAT3 dimerization.

### 2.4 SHF Inhibition of STAT3 Dimerization Targets Acetylated STAT3

**Figure 4:**
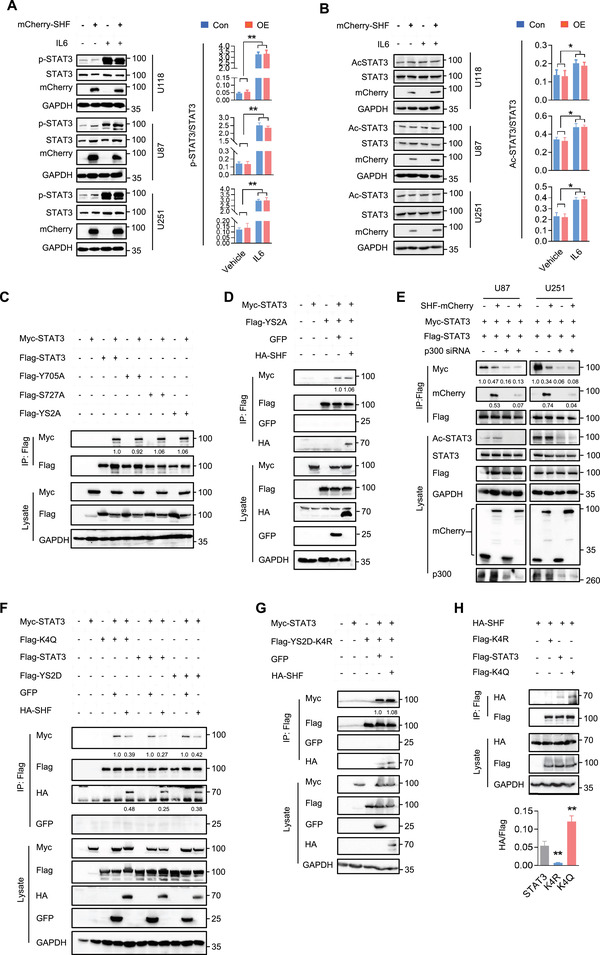
SHF inhibiting STAT3 dimerization targets on acetylated STAT3. A) Western blot analysis (left) and the statistical analysis (right) of STAT3 phosphorylation in the indicated cells (*n* = 3, Student's *t* test, **p < 0.01). Cells were treated with IL6 (10 ng mL^−1^) for 15 min before harvest. B) IP analysis (left) and the statistical analysis (right) of STAT3 overall acetylation in the indicated cells with or without IL6 (10 ng mL^−1^) stimulation for 40 min using Acetylated‐Lysine antibody (*n* = 3, Student's *t* test, *p < 0.05). C) IP analysis of STAT3 phosphorylation mutations on dimerization using Flag antibody. 293T cells were co‐transfected with wild‐type STAT3 vectors and STAT3 non‐phosphorylation point mutants (Y705A, S727A) or double point mutants (YS2A). The relative quantification of STAT3 dimerization was listed. D) The effect of SHF on the dimerization of YS2A was examined in 293T cells transfected with the indicated vectors using IP assay. The relative quantification of STAT3 dimerization was listed. E) IP assay of the effect of SHF on STAT3 dimerization in GBM cells with p300 knockdown. Indicated U87 and U251 stable cells were infected with ectopic STAT3 (Flag‐STAT3) and transfected with p300 siRNA. The relative quantification of STAT3 and SHF was listed. F) IP assay indicating the inhibition of SHF on the dimerization of phosphorylated (YS2D) or acetylated (K4Q) STAT3 in 293T cells. The relative quantification of STAT3 dimerization was listed. G) The effect of SHF on the dimerization of YS2D‐K4R was examined in 293T cells using IP assay. The relative quantification of STAT3 dimerization was listed. H) IP assay for the interaction of SHF with the indicated STAT3 construct (*n* = 3, Student's *t* test, **p < 0.01 as compared to STAT3).

Phosphorylation and acetylation are critical for STAT3 stable dimer formation upon cytokine or growth factor activation. Although SH2 domain‐containing adaptor proteins regulate several intracellular signal transduction cascades,^[^ ^13^ ^]^ SHF overexpression failed to induce detectable alterations of STAT3 phosphorylation (**Figure** **4**A) and acetylation (Figure 4B) in GBM cells, regardless of IL6 stimulation. This non‐influence is also reflected in the results of endogenous RAS/RAF/MEK signaling pathway analysis (Figure S5A, Supporting Information), and in cells with SHF depletion (Figure S5B, Supporting Information). Next, we tested whether the inhibitory function of SHF depends on phosphorylated or acetylated STAT3. To this end, the non‐phosphorylation mutants containing single site (Y705A, S727A) or dual site mutations (YS2A) at Y705 or S727 were introduced into 293T cells, followed by IP assay. Compared to wild‐type STAT3, non‐phosphorylated STAT3 at canonical sites forms a similar degree of dimerization (Figure 4C), indicating phosphorylation is a not necessary for STAT3 dimerization. Interestingly, subsequent IP analysis showed that SHF bound YS2A, but failed to impair the dimerization observed in the YS2A mutant (Figure 4D). To address whether the inhibitory function of SHF is dependent on STAT3 acetylation, siRNA mediated knockdown of p300, one major acetyltransferase regulating STAT3 acetylation,^[^ ^21^ ^]^ was applied to reduce STAT3 acetylation. As shown in Figure 4E, p300 knockdown resulted in a marked reduction of STAT3 acetylation. The IP assay indicated that STAT3 dimerization and the binding ability of SHF to STAT3 was inhibited in cells with p300 knockdown, suggesting acetylation is an essential factor for STAT3 dimerization and SHF‐binding. Accordingly, SHF‐mediated loss of STAT3 dimerization was inhibited upon p300 knockdown. To further document whether STAT3‐dimerization inhibition of SHF is targeted to a particular phosphorylated or acetylated STAT3, we established a STAT3 SH2 acetylation mimetic (K4Q) in which 4 lysine residues (K601, K615, K631 and K685) were converted to glutamine (Q), and a STAT3 phosphorylation mimic mutant (Flag‐YS2D) in which two canonical phosphorylated sites were converted to aspartate (D). YS2D or K4Q was transfected into 293T cells followed by an interaction analysis. Results demonstrated that SHF bound YS2D and K4Q, inhibiting the dimerization of these two active mimics (Figure 4F) and demonstrating that SHF targets phosphorylated or acetylated STAT3 dimerization. Since phosphorylation and acetylation are cascade events occurring after initial STAT3 activation,^[^ ^22^, ^23^ ^]^ we next asked whether SHF acts on phosphorylated or acetylated STAT3 by establishing a STAT3 phosphorylation mimic containing non‐acetylated site mutation (K4R) in which four lysine residues (K601, K615, K631 and K685) were converted to Arg (R). As shown in Figure 4G, SHF failed to inhibit the dimerization of YS2D‐K4R. Moreover, K4Q strengthened the binding ability with SHF while K4R almost lost SHF binding entirely (Figure 4H). These data demonstrate that SHF inhibits STAT3 dimerization specifically binding and inhibiting the dimerization of acetylated STAT3.

### 2.5 SHF Disrupts STAT3‐DNMT1 Interaction and Rescues Methylated Gene Expression

**Figure 5:**
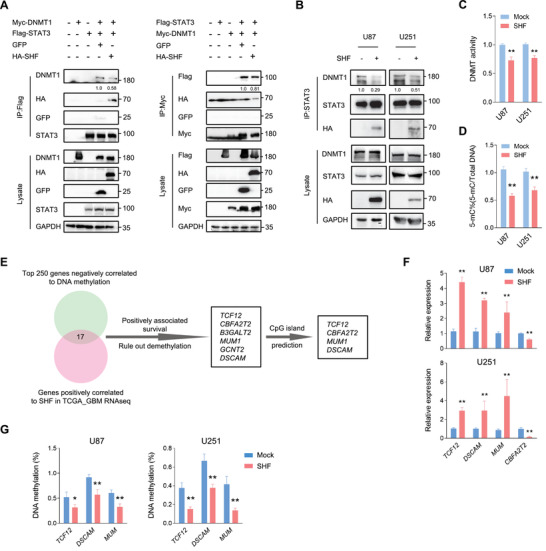
SHF disrupts STAT3‐DNMT1 interaction and reduces DNA methylation. A) Reciprocal IP analysis indicating that SHF impairs the interaction of STAT3 and DNMT1. 293T cells were transfected with the indicated vectors, followed by IP using Flag antibody (left) or Myc antibody (right). The relative quantification of STAT3/DNMT1 interaction was listed. B) IP analysis showing the effect of SHF on endogenous STAT3 and DNMT1 interaction in U87 cells. The relative quantification of STAT3/DNMT1 interaction was listed. C) SHF overexpression inhibits DNMT activity in GBM cells (*n* = 5, Student's *t* test, **p < 0.01). D) SHF overexpression reduces overall DNA methylation in GBM cells (*n* = 5, Student's *t* test, **p < 0.01). E) Schematic diagram of screening candidate genes for methylation analysis. F) qRT‐PCR analysis showing the expression changes of indicated genes induced by SHF overexpression (*n* = 4, Student's *t* test, **p < 0.01). G) qMSP assay of DNA methylation of the indicated gene promoters (*n* = 3, Student's *t* test, **p < 0.01).

STAT3 participates in the DNA methylation of tumor suppressor or anti‐apoptotic gene promoters through interaction with DNA (cytosine‐5)‐methyltransferase 1 (DNMT1).^[^ ^24^, ^25^ ^]^ Given that acetylation at K685 in the SH2 domain of SHF is crucial for binding of STAT3 to DNMT1, we explored the possibility that SHF could disrupt the interaction of STAT3 and DNMT1. A reciprocal IP analysis was performed to examine the effects of SHF on the interaction between STAT3 and DNMT1. 293T cells were co‐transfected with Myc‐DNMT1, Flag‐STAT3 and HA‐SHF or GFP plasmids. Results demonstrated that STAT3 and DNMT1 formed a complex, while SHF introduction impaired the binding between STAT3 and DNMT1 (**Figure** **5**A). Further IP assay in GBM cells indicated that SHF overexpression resulted in a reduction of the endogenous interaction between STAT3 and DNMT1 (Figure 5B). Next, we examined whether SHF inhibited the DNA methylation modification activity of DNMT1 by quantifying DNA methyltransferase activity. As shown in Figure 5C, ectopic SHF significantly inhibited the activity of DNMTs in the indicated GBM cells. As a result, the global DNA methylation of ectopic, SHF‐expressing cells was significantly lower than in control cells (Figure 5D). To further confirm the functions of SHF on DNMT1‐mediated DNA methylation, the candidate genes with a negative correlation between DNA methylation and mRNA expression in GBM were selected for the following analysis. The top 250 candidate genes were derived from MethHC (Supplementary Data 10), a database of DNA methylation and gene expression in human cancers (http://methhc.mbc.nctu.edu.tw/).^[^ ^26^ ^]^ After cross referencing genes positively correlated with SHF and those possessing overall survival significance (Figure S6, Supporting Information), seven genes were identified. Further, four candidate genes were selected following CpG island prediction analysis in the promoter (Figure 5E) using two online CpG Island prediction tools (http://www.urogene.org/cgi‐bin/methprimer/methprimer.cgi; https://sites.ualberta.ca/~stothard/javascript/cpg_islands.html). Among the four genes evaluated, TCF12, MUM1, and DSCAM mRNA expression was increased in cells expressing ectopic SHF (Figure 5F). Due to its downregulation in cells expressing ectopic SHF, CBFA2T2 was excluded from the subsequent assays. Next, methylation‐specific PCR (MSP) was applied to confirm the inhibition of SHF on DNA methylation via the DNMT1 and STAT3 interaction. SHF overexpression resulted in a marked DNA methylation reduction of targeted genes’ promoters (Figure 5G), suggesting that SHF rescues DNA methylated gene expression by disrupting the DNMT1‐STAT3 interaction.

### 2.6 The SH2 Domain is Essential for SHF Binding and Inhibition of STAT3 Dimerization

**Figure 6:**
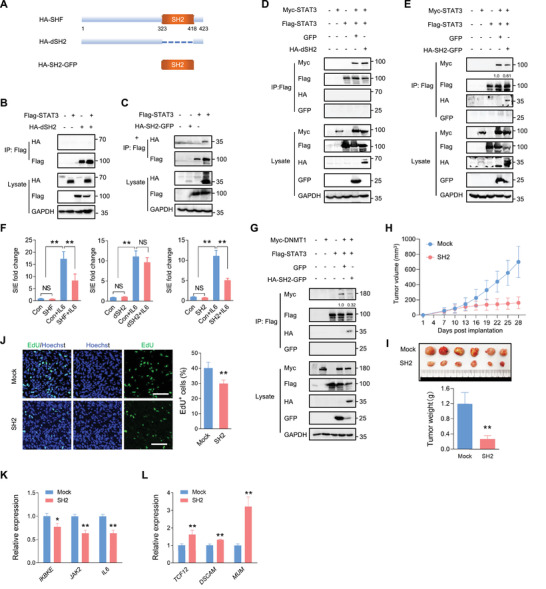
The SH2 domain is essential for SHF binding and inhibition of STAT3 dimerization. A) A schematic diagram of SHF and its deletion mutants. B) IP assay showing a SH2 deletion mutant (dSH2) fails to bind STAT3. 293T cells were transfected with the indicated vectors, followed by IP using Flag antibody. C) IP assay indicating the SH2 construct (SH2) binds STAT3. D) The effect of SH2 deletion mutant on STAT3 dimerization was examined in 293T cells transfected with the indicated vectors using IP assay. E) IP assay detecting the effect of the SH2 construct on STAT3 dimerization. The relative quantification of STAT3/DNMT1 interaction was listed. F) Luciferase assay of STAT3 transcriptional activity in the indicated cells (*n* = 8, two‐tailed Student's *t*‐test, **p < 0.01). G) IP assay detecting the effect of the SH2 construct on the interaction between STAT3 and DNMT1 in 293T cells transfected with the indicated vectors. The relative quantification of STAT3/DNMT1 interaction was listed. H) The growth curves of Mock and SH2 expressing xenograft tumors (*n* = 10, Student's *t*‐test, **p < 0.01). I) Images of excised tumors (upper) and comparison of tumors sizes (lower) at the final time point (*n* = 10, Student's *t*‐test, **p < 0.01). J) Images of EdU labeling of tumor sections (left) and statistical analysis of EdU positive cells (*n* = 10, Student's *t*‐test, *p < 0.05 **p < 0.01). K) qRT‐PCR assay measuring the expression of indicated STAT3‐targeted genes in derived tumors (*n* = 6, Student's *t* test, **p < 0.01). L) qRT‐PCR assay measuring the expression of indicted methylated genes in derived tumors (*n* = 6, Student's *t* test, **p < 0.01).

Since SHF is a protein containing a SH2 domain, we sought to determine the involvement of the SH2 domain in mediating STAT3 dimerization inhibition and tumor suppression. To this end, we performed IP experiments using a SH2 construct and SH2 deletion mutant based on the known SHF protein structure (**Figure** **6**A). Compared with full‐length SHF, SHF without the SH2 domain completely lost STAT3‐binding capacity (Figure 6B). In contrast, the SH2 construct alone could bind STAT3 without the aid of any other domains (Figure 6C), indicating that the SH2 domain is essential and sufficient for SHF binding of STAT3. Consistent with STAT3‐binding deficiency, the SH2 deletion mutation failed to inhibit STAT3 dimerization (Figure 6D; Figure S7A, Supporting Information) or to impact STAT3 transcriptional activity either under cytokine‐free conditions or in the presence of IL6 (Figure 6F). On the contrary, the SH2 construct inhibited STAT3 dimerization (Figure 6E; Figure S7B, Supporting Information) and consequently inhibited STAT3 transcriptional activity (Figure 6F). Subsequently, the SH2 construct acted as an inhibitor of STAT3 and DNMT1 interaction (Figure 6G; Figure S7C, Supporting Information). Next, we explored whether the SH2 construct functioned as a tumor suppressor like SHF. The subcutaneous tumor model indicated that tumors derived from U87 cells expressing SH2 grew more slowly than tumors derived from cells containing Mock (Figure 6H), eventually forming much smaller tumors (Figure 6I). The tumor inhibition action of SH2 was also corroborated by EdU incorporation assay (Figure 6J). Moreover, SH2 expression inhibited STAT3 targeted gene expression (Figure 6K) and promoted the expression of methylated genes in the derived tumors (Figure 6L). Collectively, these results indicate that the SH2 domain is essential for SHF binding on STAT3, performing the STAT3 inhibitory functions of the entire protein.

### 2.7 A Peptide Derived from the STAT3‐Binding Sites of SHF is an Effective GBM Suppressive Strategy

**Figure 7:**
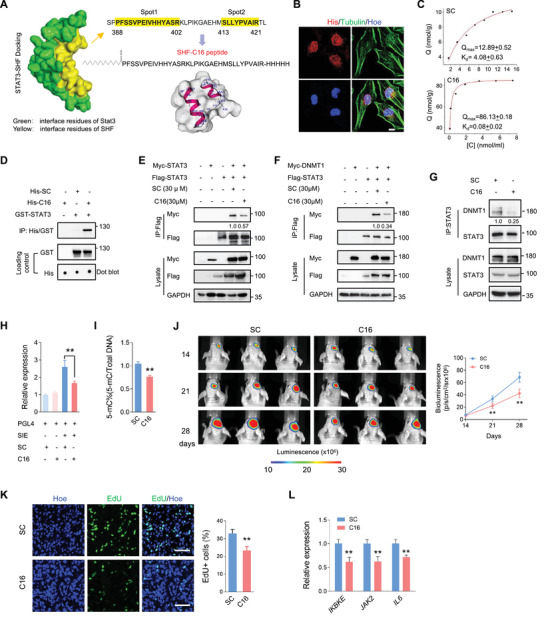
Peptide derived from the STAT3‐binding sites of SHF is an effective GBM suppressive strategy. A) Binding model between SHF and STAT3 using ZDOCK server. The suggested interactive interface for SHF included two sites (yellow). B) IF staining determined the cell penetrability and nuclear‐localization of C16. U251 cells were incubated with C16 or SC for 2 h and performed IF staining using the indicated antibodies. Bars, 10 µm. C) The statical analysis of adsorption equilibrium assay detecting the binding of C16 and SC with recombinant STAT3. D) Pull‐down assay indicating C16 binds STAT3. GST‐STAT3 was incubated with C16 or SC for 2 h, and the pull‐down assay was performed using Anti‐His magnetic beads. E) IP assay showing the effect of C16 on STAT3 dimerization. 293T cells transfected with indicated vectors were incubated with C16 for 2 h. The relative quantification of STAT3 dimerization was listed. F) IP assay detecting the effect of C16 on the interaction of STAT3 and DNMT1. 293T cells expressing ectopic STAT3 and DNMT1 was incubated with C16 for 2 h. The relative quantification of STAT3/DNMT1 interaction was listed. G) IP assay indicating C16 inhibits the endogenous interaction of STAT3 and DNMT1. U87 cells were incubated with C16 for 2 h and followed with an IP assay (*n* = 3, Student's *t* test, **p < 0.01). The relative quantification of STAT3/DNMT1 interaction was listed. H) Luciferase assay of STAT3 transcriptional activity in U87 cells incubated with C16 for 12 h (*n* = 3, Student's *t*‐test, **p < 0.01). I) Global DNA methylation assay of U87 cells treated with C16 (*n* = 3, Student's *t* test, **p < 0.01). J) Bioluminescent images of intracranial xenografts derived from the implantation of the indicated cells (left) and statistical analysis of tumor volume (right; *n* = 6, Student's *t*‐test, **p < 0.01). K) Representative EdU staining images of xenograft tumor sections (left) and the statistical analysis of the fraction of EdU‐positive cells (right). Positive cells were quantified 20 randomly selected fields per mouse (*n* = 6, Student's *t*‐test, **p < 0.01). Scale bars, 50 µm. L) qRT‐PCR analysis detecting the expression of indicated STAT3‐targeted genes in derived tumors (*n* = 6, Student's *t* test, **p < 0.01).

Considering that the SH2 domain predominantly represents the inhibition of SHF on STAT3 dimerization and activity, we speculated that this domain may be an optional target for GBM suppression. To this end, we analyzed the mechanical interaction of SHF and STAT3. The structures of human SHF (aa265‐421) and STAT3 (aa130‐715). both of which spanned their respective SH2 domain. were simulated by Swiss‐Model server (https://swissmodel.expasy.org/). To simulate the interaction of SHF and STAT3, Discovery studio 2018 was employed to complete protein‐protein docking using ZDOCK as an initial stage. A rigid body molecular docking algorithm utilizing a fast Fourier transform (FFT) algorithm was used to further improve performance for searching in translational space. All the available structures from PDB were used to calculate the docking poses and the structures obtained were subjected to energy minimization filtration using the smart minimize algorithm (Max steps 200, RMS gradient 0.01). The highest resulting ZDock score was used as the appropriate conformational pose. Regarding ZDOCK models, the suggested interface for SHF included two sites spanning the amino acids 388–402 and 413–421 (**Figure** **7**A). Since these two spots are located closely to one another, the sequence spanning amino acids 388–421 was used as a peptide design master. To obtain a peptide with high internalization efficiency and a strong half‐life, we introduced a palmitic acid at the N‐terminus of the master sequence and amidated the C‐terminus. The designed peptide was named C16. A scrambled peptide containing a scrambled sequence targeting Spot1 was named SC and served as a negative control peptide. C16 was added to culture medium for 48 h and the immunofluorescence staining using His‐antibody determined the cell penetration and nuclear localization capacity of C16 (Figure 7B). The static adsorption equilibrium experiment of immobilized STAT3 peptide was used to detect the binding rate of the synthesized peptide with STAT3 (Figure 7C). The concentration of immobilized STAT3 used was 85 nmol g^−1^ wet gel. The results showed that the maximum static adsorption of C16 was 86.13 ± 0.18 nmol g^−1^ wet gel and the equilibrium dissociation constant KD was 0.08 ± 0.02 nmol mL^−1^. While the maximum static adsorption of the SC peptide was 12.89 ± 0.52 nmol g^−1^ wet gel, the equilibrium dissociation constant KD was 4.08 ± 0.63 nmol mL^−1^. The equilibrium dissociation constant KD of C16 was two orders of magnitude lower than that of the SC peptide, indicating a significant targeting affinity. Its maximum value of static adsorption was consistent with the actual protein amount of immobilized STAT3, confirming to the 1:1 interaction model. The in vitro IP analysis confirmed that C16 contains the STAT3 binding capacity (Figure 7D). Subsequently, IP assay (Figure 7E), as well as FRET assay (Figure S8, Supporting Information), showed that C16 effectively inhibits STAT3 dimerization. C16 addition also resulted in a decrease of ectopic (Figure 7F) or endogenous (Figure 7G) STAT3/DNMT1 interaction. Additionally, C16 addition in U87 cells resulted in a significant decrease of STAT3 transcriptional activity (Figure 7H) and global DNA methylation (Figure 7I). Thus, C16 acted as a powerful inhibitor of STAT3 dimerization and associated activity. Next, we determined the implication of C16 in tumor inhibition. As shown in Figure S9A, Supporting Information, cell growth assay indicated that C16 inhibited cell growth with concentration dependence in U87 and U118 cells. A concentration of 25 um was determined to be adequate for use in the subsequent experiments. As expected, C16 effectively impaired colony growth and promoted chemosensitivity without increasing spontaneous apoptosis (Figure S9B,C, Supporting Information). Further, the impact of C16 on tumor propagation was examined in an intracranial xenograft model. U87 cells expressing luciferase were implanted into mouse brains. The mice were injected intraperitoneally (i.p.) with C16 or SC once every 2 days from the third day after cell transplantation. Bioluminescent analyses showed that C16 treatment potently inhibited the growth of intracranial tumors (Figure 7J). To investigate the effect of C16 on cell proliferation, EdU was injected 24 h prior to sacrifice. The immunofluorescent analyses of derived tumor sections revealed that EdU positive cells were significantly decreased in the xenografts treated with C16 (Figure 7K). Moreover, qRT‐PCR analysis of the derived xenografts indicated that STAT3 targeted genes were significantly down‐regulated (Figure 7L). Collectively, these data demonstrate that C16 derived from hot spots of SHF‐bound STAT3 is a promising therapeutic strategy for GBM inhibition.

## 3 Discussion

It's well established that STAT3 is a critical mediator of tumorigenesis, tumor progression, and suppression of anti‐tumor therapy in GBM.^[^ ^2^ ^]^ Here, we identify the SH2 containing adaptor protein, SHF, which acts as a tumor suppressor in GBM via a novel mechanism involving STAT3 regulation. SHF inhibits STAT3 dimerization and STAT3‐DNMT1 interaction, resulting in a decrease in STAT3 transcriptional activity and de‐methylation of the promoter of target genes. The SH2 domain was demonstrated to be essential for SHF binding and inhibiting STAT3 dimerization, completely emulating the functions of SHF on STAT3. The significance of this study provides a novel peptide derived from hot‐spots of SHF‐bound STAT3, suggesting candidacy as a therapeutic approach for GBM.

STAT3 has been extensively studied as a therapeutic target in cancers, and STAT3 inhibitors or drugs have been greatly progressed.^[^ ^27^, ^28^ ^]^ Two major solutions including targeting upstream regulators and blocking phosphorylation, dimerization, nuclear translocation, and DNA binding^[^ ^27^, ^28^, ^29^ ^]^ have been extensively studied. However, to date, no STAT3 inhibitor has been approved for cancer therapy. Among the activation process, the dimerization is a vital step for target DNA binding and transcription initiation. Considering dimerization is the final process in STAT3 activation, antagonizing STAT3 dimerization is the preferred therapeutic target.^[^ ^27^ ^]^ Consequently, inhibitors targeting the SH2 domain of STAT3 have been extensively developed for blocking dimerization and DNA‐binding directly. Among these, small‐molecule inhibitors antagonizing STAT3‐SH2 have shown potential clinical application.^[^ ^5^, ^7^, ^8^, ^10^ ^]^ STAT3 dimerization can occur in the absence of Tyr705 phosphorylation,^[^ ^30^ ^]^ and unphosphorylated STAT3 (U‐STAT3) also exerts transcriptional regulation on certain genes which are not targets of phosphorylated STAT3.^[^ ^31^, ^32^ ^]^ Moreover, STAT3 can be acetylated at both the N‐terminal (K49 and K87) and the SH2 domain (K685 and K631), two modifications considered to be required for proper nuclear localization, transcription, and DNA methylation.^[^ ^3^ ^]^ The most well studied STAT3 acetylation occurs on the K685 residue, a modification critical for the stable dimerization of STAT3. Acetylation on K685 plays vital roles for U‐STAT3 target gene regulation,^[^ ^6^, ^33^, ^34^ ^]^ indicating acetylation regulated STAT3 activity is independent of phosphorylation. To some extent, acetylation it is more important for STAT3 function than phosphorylation. However, using the K4R mutant spanning K685R, the current study found that unacetylated STAT3 can form a dimer. Although acetylation modification at other sites cannot be excluded, at least, this discovery reveals the complexity and diversity of STAT3 dimerization regulation. To date, the roles of U‐STAT3 and non‐acetylated STAT3 in regulating the actions of STAT3 are far from clear. This may also be one of the reasons why STAT3 has dual functions in cancer promotion and inhibition.^[^ ^35^, ^36^, ^37^ ^]^ Thus, it is not difficult to predict the successful clinical application of small‐molecule inhibitors targeting STAT3 dimerization.

One novel finding of the current study was that SHF binds STAT3 and selectively blocks acetylated STAT3 dimerization. Although the binding of STAT3 covers K685, SHF failing to inhibit STAT3 acetylation raises two interesting concerns of how SHF acts on acetylated STAT3 dimerization and why SHF has no effect on non‐acetylated STAT3 dimerization. A recent study reported that there is no structural difference between acetylated and non‐acetylated STAT3,^[^ ^23^ ^]^ which may rule out the possibility that SHF alters the structure of acetylated STAT3. To date, there is no report documenting the difference between acetylated and non‐acetylated STAT3 in the context of dimerization. Due to the finding that STAT3‐binding of SHF is acetylation dependent, it's reasonable to believe that acetylation is enough to determine the protein binding status of STAT3, and that SHF may occupy the binding site of acetylated STAT3 with other proteins. A limitation of the current study is that only four lysine sites located in the SH2 domain of STAT3 were used to examine the effect of SHF on STAT3 dimerization. K685 is the most studied acetylation modification of STAT3, acting as a required modulator of STAT3 dimer formation and subsequent transcriptional regulation.^[^ ^6^ ^]^ However, besides K685 and the other three sites, two lysine residues (K49 and K87) in the NH2‐terminus region and two lysine residues (K707 and K709) in the C‐terminus are also acetylated sites in STAT3.^[^ ^3^, ^38^ ^]^ The function of these modifications is currently debated, while the importance of acetylation to STAT3 is consistent.^[^ ^3^ ^]^ The provided evidence that K685R can dimerize indicates that K685 is not the only acetylation site that determines STAT3 dimerization. SHF has no effect on overall STAT3 acetylation, excluding the possibility that SHF affects STAT3 dimerization and activity by modulating acetylation of other sites. It supposed that the effect of SHF on STAT3 is mainly aimed at the dimerization mediated by acetylation in SHF‐binding region of STAT3. The significance of acetylation in this region to STAT3 function needs to be clarified.

In addition to homodimerization, STAT3 also forms heterodimers with other STATs, followed by translocation to the nucleus where it can mediate unique functional events.^[^ ^20^, ^39^, ^40^ ^]^ STATs share structural similarities and conserved functional regions, among which the SH2 domain mediates homo‐ and heterodimerization of STAT monomers during activation. For STAT3, the common heterodimer binding proteins are STAT1 and STAT5.^[^ ^20^ ^]^ STAT3 and STAT5 are currently considered oncogenes, while STAT1 appears to play opposite roles in tumorigenesis.^[^ ^40^ ^]^ One previous study reported that STAT3 does not suppress STAT1 tyrosine phosphorylation or nuclear translocation but instead sequesters STAT1 and suppresses the formation of DNA‐binding STAT1 homodimers,^[^ ^41^ ^]^ suggesting STAT3 and STAT1 mutually inhibit each other through heterodimer formation. However, the current data reveals that no heterodimer formation of STAT3 and STAT1 occurs in an environment without inflammatory factors in GBM cells. Although binding STAT1, SHF fails to alter the interaction between STAT3 and STAT1 without external stimulation. It suggests that tumor inhibition of SHF probably underlies the regulation of STAT1 independent of STAT3, a question worthy of further exploration. Moreover, the current finding that SHF inhibits STAT3/DNMT1 interaction provides a clue that SHF also acts on other STAT3 interacting proteins, especially those binding via the STAT3 SH2 domain such as gp130, leukemia inhibitory factor receptor (LIFR), epidermal growth factor receptor (EGFR), interleukin 10 receptor (IL‐10R), etc.^[^ ^39^ ^]^ The interaction between EGFR and STAT3 occurs in the nucleus upon ligand‐dependent EGFR activation.^[^ ^42^ ^]^ Given that SH2 containing proteins can dock with phosphorylated tyrosine residues on EGFR,^[^ ^43^ ^]^ SHF is expected to be a impedance of EGFR/STAT3 complex formation in the nucleus. As an SH2 adaptor protein of putative tyrosine phosphorylation sites,^[^ ^16^, ^17^ ^]^ SHF is potentially involved in signal transduction, and is itself regulated by phosphorylation. Additionally, other protein posttranslational modifications, including sumoylation, acetylation, ubiquitination, and so on may similarly modulate protein interactions, cellular localization, and functions of SHF.

In the current study, we demonstrate the application of C16 peptide in targeting the SHF/STAT3 interaction in GBM. The core sequence of C16 is only 34 amino acids, which binds a limited surface of STAT3. Among the four acetylation sites of the STAT3 SH2 domain, it only convers K685, suggesting that its inhibition on STAT3 is K685 acetylation dependent. The critical roles of K685 acetylation for STAT3 stable dimerization and DNA binding is largely recognized,^[^ ^3^ ^]^ indicating that C16 is a specific nuclear STAT3 gene inhibitor. The current evidence derived from STAT3 dimerization and methylation inhibition to in vivo tumor suppression make C16 an attractive strategy for further development and utilization, such as improving solubility, bioavailability, and tumor‐targeted delivery. Another application direction is to degrade acetylated STAT3 by using PROTAC and other technologies. Using C16 as the medium for sequestering acetylated STAT3 using design proteolysis targeting chimera (PROTAC) STAT3 degraders^[^ ^7^ ^]^ is regarded as one of the directions for future improvement.

In conclusion, this study identifies SHF as a new tumor suppressor for GBM treatment. By blocking acetylated STAT3 dimerization and DNMT1 interaction SHF may serve as an attractive therapeutic target for patients with a range of cancers, not only GBM. The derived therapeutic peptide also deserves further exploration.

## 4 Experimental Section

### Online Cancer Database Analysis

The expression of genes coding the SH2 domain‐containing adapter protein was assessed between normal and GBM‐affected brains using the TCGA‐GBM database accessible through UALCAN (http://ualcan.path.uab.edu/; Supplementary Data 1). A large cohort expression and survival analysis emphasizing SHF mRNA expression was performed using the glioma database found on the GlioVis data portal (http://gliovis.bioinfo.cnio.es/; Supplementary Data 2). The clinical characteristics of the cohort and SHF expression data are described in Supplementary Data 3. For the gene set enrichment analysis (GSEA), the software available on the GenePattern online server (http://www.gsea‐msigdb.org/) GSEA v4.1.0 and the Molecular Signatures Database (MSigDB database v7.4) were used. Overlaps of SHF correlated gene sets (metric for ranking genes) were computed according to the specified categories in MSigDB collections using the default setting. Pearson was selected in the Metric for ranking genes parameter.

### Clinical Sample and Immunohistochemical Staining

SHF protein expression in GBM tissue was analyzed using immunohistochemical (IHC) staining on a tissue array. The tissue microarray chips contained a total of 64 samples (58 GBM and 5 normal brain) with follow‐up data obtained from the affiliated hospital. All patient information was obtained and used in accordance with approved protocols from the institutional review boards of the participating institutions (No.(2020)353). The clinical information of the cohort is described in Table S1, Supporting Information. Briefly, tissue slides were incubated with rabbit anti‐SHF antibody (1:4000; Sigma‐Aldrich, St. Louis, MO), anti‐rabbit secondary antibody from Zymed Systems (Invitrogen, Carlsbad, CA) and 3,3’‐diaminobenzidine to visualize the IHC labeling. Slides were counterstained lightly with crystal violet. Normal rabbit IgG was used to confirm the specificity of the IHC. SHF expression was scored semi‐quantitatively based on an established immunoreactivity scoring (IRS) system, with some modifications.^[^ ^19^ ^]^ Briefly, IRS was calculated as the product of the proportion score (%) multiplied by the staining intensity score (0–3). The proportion score represented the percentage of positive cells while the intensity score represented the average intensity of staining (0: no staining; 1: yellow, 2: claybank; and 3: tawny). Two blinded pathologists reviewed and scored the slides. The mean IRS score was considered as the final IRS (Supplementary Data 4).

### Cell Lines, Primary Cell Preparation, and Culture Conditions

The human glioma cell lines U87, U118, U251, T98G, A172, H4, and human embryonic kidney cell line, 293T, were obtained from the Cell Bank of Type Culture Collection of the Chinese Academy of Sciences (Shanghai, China). All cells were cultured in DMEM with 10% fetal bovine serum (FBS; Invitrogen, Carlsbad, CA). These cells were characterized by Genewiz, Inc. (China) using short tandem repeat markers and were confirmed to be mycoplasma‐free. For 3D‐Spheroid culture, 400 cells were seeded in spheroid microplate (Corning Inc., NY, USA) according to a 3D‐spheroids formation and growth protocol. The spheroids were cultured with GBO medium with a minor modification (without insulin) according to a previously published report,^[^ ^44^ ^]^ changing half of the medium every 2 days. For patient‐derived GBM cell culture, fresh GBM tissues derived from surgery were collected immediately after tumor resection, washed, minced, and enzymatically dissociated. Tumor cells were resuspended and cultured in GBO medium to allow tumor sphere formation. The primary spheres were cultured for 10 days to obtain enough cells for passage. The third or fourth passage of cells was used for subsequent experiments, including tumor xenografts and Western blot assay.

### Cell Growth and Colony Formation Assay

Cell growth was quantified using the Cell Counting Kit‐8 Assay Kit (Bimake, Houston, TX). Each experiment was repeated at least six times. For the colony formation assay, 800 cells were seeded into each well of a six‐well plate with soft agar (Agarose; Sigma, St. Louis, MO) and maintained in a medium containing 10% FBS for 14 days. The colonies were fixed with methanol and stained with 0.1% crystal violet; the clones containing at least 50 cells were counted using an inverted microscope.

### EdU Cell Proliferation Assay

Cell proliferation and DNA replication was analyzed by EdU incorporation method as described previously.^[^ ^45^ ^]^ Briefly, cells were cultured in 24‐well plate for 36 h followed by an incubation of 10 ≤ µm EdU (KeyGen Biotech, China) for 2–3 h and fixation with 4% paraformaldehyde (PFA). The staining procedure was performed according to the manufacturer's instructions for the kFluor488 Click‐iT EdU Kit (KeyGen Biotech, China). After staining, the coverslips were mounted with Gelmount containing Hoechst 33 342 (Sigma) and the cells in 60 mm dish were analyzed by a FACScan flow cytometry (BD, FACSCanto II, CA, USA).

### In vitro Invasion Assay

Cell invasion assays were performed using transwell chambers (8‐mm pore size, Millipore, Billerica, MA, USA) coated with Matrigel (15 mg mL^−1^; BD bioscience, Bedford, MA). A total of 1 × 10^5^ cells in 200 µL of the culture medium (supplemented with 0.1% FBS) were added into the upper chamber, and 600 µL of medium supplemented with 10% FBS were added into the lower chamber to serve as a chemotactic agent. After 48 h of incubation, cells remaining in the upper chamber were carefully cleared, and cells invading into the lower chamber were fixed and stained with crystal violet. The stained cells were dissolved by 3% glacial acetic acid; the number of invading cells was quantified by detecting the absorbance of the solution at OD450.

### 3D‐Spheroid Formation and Growth Measurements

Tumor sphere formation was analyzed as described previously, with some modification.^[^ ^19^ ^]^ Briefly, the growth of the 3D‐Spheroid cultured cells was monitored by a microscope with a real‐time camera (EVOS FL Auto Imaging System, Life Technologies, Carlsbad, CA, USA). For sphere growth assay, photographs of tumor spheres were taken at the indicated time points and the sphere area was measured. The sphere growth was expressed as the ratio of the area at different time points relative to the area at 1 day.

### Cell Apoptosis Analysis

Cell apoptosis was detected by flow cytometry according to the manufacturer's instructions of the Annexin V‐FITC Apoptosis Detection Kit (KeyGen Biotech, China). Briefly, cells were incubated with Doxorubicin (DOX, 2 µm) for 48 h. Cells were harvested and rinsed with ice‐cold PBS and then resuspended in 200 µL of binding buffer. 10 mL of AV‐FITC stock solution was added to cell suspensions and incubated for 15 min at RT. Cells were further rinsed with 200 µL ice‐cold PBS and immediately analyzed by a FACScan flow cytometry.

### STAT3 Transcriptional Reporter Assay

STAT3 transcriptional activity was evaluated by a luciferase reporter system according our previously study.^[^ ^19^ ^]^ Briefly, cells were transfected with the SIE reporter plasmid (Promega, Madison, WI). The pRL‐TK Renilla luciferase plasmid (Promega) was also transfected as an internal control reporter. 24 h after transfection, cells were treated with or without IL‐6 (20 ng mL^−1^; Peprotech Inc., Rocky Hill, NJ) for 4 h. Cells were collected, lysed, and measured using the Dual‐Glo Luciferase Assay System kit (Promega).

### Vector Construction and Transduction

Full‐length cDNA encoding human SHF, STAT3, and DNMT1 were amplified by PCR and verified by DNA sequencing. The mCherry‐SHF lentivirus was constructed by inserting the cDNA sequence into lentivirus vector GV348 (Genechem, Shanghai, China) with a mCherry‐tag. HA‐SHF was constructed by inserting cDNA into expression vector GV219 with a HA tag. The SH2 domain plasmid and deletion mutant were constructed based on the wild‐type SHF plasmid. STAT3 plasmids were constructed by inserting cDNA into expression vector GV141 with a Flag or Myc tag. The STAT3 non‐phosphorylation mutants (Y705A, S727A, YS2A), in which Y705 or/and S727 were replaced with alanine (A), were created from the wild‐type STAT3 plasmid. A STAT3 a phospho‐mimetic (YS2D), in which Y705 and S727 were replaced with aspartic acid (D), was also produced. The SH2 domain acetylation mimetic (K4Q) and non‐acetylation mutant (K4R) were constructed based on the STAT3 vector with lysine (K) mutations in the SH2 domain. Specifically, the lysine (K) mutation was altered to either a glutamine (Q) or arginine (R) at sites K601, K615, K631, and K685. A phospho‐mimetic with a SH2 non‐acetylation mutant (YS2D‐K4R) was constructed based on the YS2D vector. SHF‐DsRed and STAT3‐DsRed were constructed by inserting cDNA into expression vector GV147 with a DsRed2 tag (Genechem). STAT3‐EGFP was constructed by inserting cDNA into expression vector GV230 with a EGFP tag (Genechem). STAT3‐nEGFP, STAT3‐cEGFP, and SHF‐cEGFP were constructed by inserting cDNA into expression vector GV712 (Genechem) with a N‐EGFP (amino acids 1–172 of EGFP N‐terminal) or C‐EGFP (amino acids 155–238 of EGFP C‐terminal) tag. Finally, the DNMT1 expression plasmid was constructed by inserting cDNA into the expression vector GV219 with a Myc tag.

### Double Immunofluorescence Staining

Double immunofluorescence (IF) staining was performed according to our previously established protocol.^[^ ^19^ ^]^ Antibodies used to determine the indicated protein are shown in Table S2, Supporting Information. Cell nuclei were counterstained with Hoechst 33 342 and sections were subsequently washed, mounted, and examined using the Olympus BX60 light microscope (Olympus, Center Valley, PA, USA) or a laser scanning confocal microscope (Leica Microsystems GmbH, Mannheim, Germany). The specificity of the immunofluorescent labeling was confirmed by primary antiserum omission and normal mouse/rabbit and donkey serum controls.

### Western Blot Assay

Standard Western blot assays were used to measure protein expression. Antibodies used to determine the indicated proteins are specified in Table S2, Supporting Information. The uncropped original images are also shown in Figure S5, Supporting Information.

### Immunoprecipitation Assays

Immunoprecipitation (IP) experiments were performed to analyze the protein interactions. In brief, cells were transfected with the indicated plasmids and whole‐cell lysates were precipitated using Protein A/G beads (Santa Cruz, CA) with the indicated antibodies. Precipitated products were analyzed by Western blot using the specified antibodies. Antibodies used in IP and Western blot experiments are shown in Table S2, Supporting Information.

### Reverse‐transcription Quantitative PCR

Total RNA was extracted using TRIzol reagent (Invitrogen) according to the manufacturer's instructions. cDNA was synthesized with the M‐MLV Reverse Transcriptase Kit (Cwbio, Beijing, China). PCR analyses were conducted to quantify mRNA expression using Real SYBR Mixture (Cwbio) on a Lightcycler 480 II instrument (Roche Applied Science). GAPDH severed as an internal control. The specific oligonucleotide primer pairs are listed in Table S3, Supporting Information.

### ChIP and ChIP‐qPCR Assays

The ChIP assay was performed using the SimpleChIP Enzymatic Chromatin IP Kit (Cell Signaling, Inc., Danvers MA) according to the manufacturer's instructions. Briefly, 2 × 10^7^ cells and 5 µg anti‐STAT3 antibody were used for each ChIP. The mouse IgG and rabbit anti‐Histone H3 antibody was applied as negative control and positive control, respectively. After reversal of cross‐links and DNA purification, the samples were used for quantitative real‐time PCR with specific primers targeting gene promoters (Table S4, Supporting Information). ChIP efficiency was expressed according to our previously established method.^[^ ^19^ ^]^

### Recombinant Protein Design

To achieve an optimal targeting strategy for STAT3 inhibition of SHF, more precise identification of the STAT3, SHF binding site was analyzed. Briefly, the structures of human SHF (aa 265–421) and STAT3 (aa 130–715) spanning the SH2 domain were simulated by Swiss‐Model server (https://swissmodel.expasy.org/), using Mus musculus STAT3 (1BG1) and Homo sapiens Tyrosine‐protein kinase ABL1 (6AMW) as templates, respectively. To simulate the interaction of SHF and STAT3, Discovery studio 2018 was employed to complete protein‐protein docking using ZDOCK (http://zdock.umassmed.edu/) as an starting point. All the available structures from Protein Data Bank (PDB; https://www.rcsb.org/) were used to calculate the docking poses and the structures obtained were subjected to energy minimization using the smart minimize algorithm (Max steps 200, RMS gradient 0.01). The resulting ZDock scores with the highest values were used as appropriate conformational poses. The resulting interface for SHF included two closely located sites. The sequence spanning these two sites was used as a peptide design master. To obtain a peptide with high internalization efficiency and a strong half‐life, we introduced a palmitic acid at the N‐terminus of the master sequence and amidated the C‐terminus. To facilitate screening and identification, 3× His tags were added to the C‐terminal. The designed peptide was named C16 and synthesized directly (Genscript, China). A scrambled peptide (SC) containing the same flanking sequence with a scrambled sequence (PASSNCEIVPMYHIRKLPIKGAEHMSLLYPVAIR) targeting Spot1 of the master sequence was designed and used as a negative control.

### C16 Synthesis

The synthesis of C16 was accomplished using a solid‐phase methodology on Fmoc Rink Amide‐MBHA resin (GL biochem, Shanghai, China) by a microwave synthesizer (CEM, NC, USA). Fmoc‐protected amino acids (GL Biochem, Shanghai, China) were used. After the quantified resin was swelled, deprotected, and washed, a mixed solution of Fmoc‐protected amino acid and activator (HBTU, HOBT, and DIPEA, all purchased from GL biochem, Shanghai, China) was added. Then the mixture was bubbled with nitrogen under microwave irradiation and washed with DMF (Samsung fine chemicals, Korea). The procedures of deprotection and coupling were repeated with the relevant Fmoc‐protected amino acids to create the peptide‐resin complex. Then resin was washed successively with DMF three times. The final peptide was cleaved using TFA/TIS/H2O (95:2.5:2.5, v/v/v) for 4 h at room temperature and precipitated with cold ethyl ether. Then ethyl ether was removed through centrifugation. All peptides were purified by preparative reversed‐phase high‐performance liquid chromatography (RP‐HPLC; Shimadzu LC‐10) on a C18 column (5 µm, 340 × 28 mm). The purity of C16 was demonstrated above 95%.

### Static Adsorption Equilibrium Assay

Adsorption equilibrium experiments were carried out to explore the adsorption behavior of C16 by reconstituting 0.755 mg (powder) of STAT3 into flasks containing 20 mL of PBS solution mixed with different concentrations of C16 and scramble (2, 4, 6, 8, 10, 12, 14, 16, 18, 20 µm, respectively). The mixture was stirred constantly for 12 h until complete adsorption of C16 and scramble from STAT3. The free C16 and scramble peptide concentration in the PBS solution was evaluated using NanoDrop 2000 by measuring absorbance intensity at *λ*_max_ = 240 nm. The Langmuir isotherm equation was applied to identify the *Q*_max_ (nmol g^−1^) and *K*_d_ (nmol mL^−1^) of C16.

(1) $$Q=\frac{Q_{\max}\left[C^{*}\right]}{K_{d}+\left[C^{*}\right]}$$

### Global DNA Methylation Assay

Global DNA methylation status was quantified by the MethylFlash Global DNA Methylation (5‐mC) ELISA Easy Kit (Epigentek, Brooklyn, NY). Levels of 5‐methylcytosine (5‐mC) were calculated according to the manufacturer's instructions. In brief, the purified and quantified genomic DNA were bound to strip‐wells treated for high DNA affinity. The methylated fraction of the DNA was detected using capture and detection antibodies. Reaction was then quantified colorimetrically by reading the absorbance in a microplate spectrophotometer. The percentage of methylated DNA was proportional to the OD intensity measured.

### DNMT Activity Assay

Global DNA methyltransferase activity was measured using the EpiQuik DNMT (DNA Methyltransferase) Activity/Inhibition Assay Ultra Kit (Epigentek). In this assay, the unique cytosine‐rich DNA substrate is stably coated on the strip wells. Cell nuclear protein was extracted and incubated with the substrate. The methylated DNA can then be recognized with an anti‐5‐methylcytosine antibody. The ratio or amount of methylated DNA, which is proportional to enzyme activity, was colorimetrically quantified through an ELISA‐like reaction.

### Quantitative Methylation‐specific PCR Assay

Genomic DNA from U87 control cells or cells expressing ectopic SHF were isolated using Universal Genomic DNA kit (Cwbio) and quantified using NanoDrop (Thermo Fisher Scientific, Leicester, UK). Bisulfite converted DNA was used to perform the qMSP by Methylamp MS‐qPCR Fast Kit (Epigentek, Brooklyn, NY) according the manufacturer instructions. For each reaction, 20 ng bisulfite‐treated DNA was used as a template. Primers for the genes of interest were designed using MethPrimer (http://www.urogene.org/methprimer/index.html; Table S5, Supporting Information). ACTB (*β*‐actin) gene was used as a reference gene. No‐template controls were included in each run as negative controls. An EpiTect Control DNA, a 100% methylated DNA (Qiagen, Hilden, Germany), was used as a positive control for all genes studied. The percentage of methylated reference value (PMR) was calculated by dividing the gene/ACTB ratio in a sample by the gene/ACTB ratio in the EpiTect Control DNA (Qiagen) sample and multiplied by 100. Parallel PCR reactions were carried out for the genes of interest and reference. PMR values were detected using the comparative CT method. The relationship between the percentages of methylated DNA molecules and CT is described as PMR = 2^−ΔΔ^ *^CT^* × 100%.

### Fluorescence Resonance Energy Transfer Assay

Spectral fluorescence resonance energy transfer (FRET) assay was used to examine the dimerization of STAT3 and interaction between STAT3 and SHF. The procedure was performed as described previously.^[^ ^46^ ^]^ Briefly, U251 cells were seeded at a density of 20 000 cells/compartment on coverslips coated with 1% poly‐l‐lysine (Sigma) in 24 well‐plates. For detecting SHF inhibiting STAT3 dimerization, ectopic SHF‐expressing stable U251 cells were transfected with STAT3‐DsRed and STAT3‐EGFP. For detecting SHF binding STAT3, U251 cells were transfected with SHF‐DsRed and STAT3‐EGFP. Cells were imaged on a Leica TCS SP8 confocal microscope (40× objective and 10× objective, 488 nm Argon laser line) after 24 h. Cells were excited with 488 nm laser (donor), and the emission of the acceptor DsRed was recorded at 565 nm. The FRET efficiency was calculated on a pixel‐by‐pixel basis with the following equation: FRET efficiency = FRETcorr / (FRETcorr + Donor). “FRETcorr” is the pixel intensity in the corrected FRET image and “Donor” is the intensity of the corresponding pixel in the donor channel image. To increase the reliability of the calculations and prevent noise from distorting the calculated ratio, we excluded pixels below 50 intensity units and saturated pixels from the calculations and set their intensities to zero. These pixels are shown in black in the pseudo‐colored FRET efficiency images. An exact measurement of the concentration of proteins participating in the interaction was beyond the scope of this study.

### Bimolecular Fluorescence Complementation Analysis

To directly visualize STAT3‐SHF interactions in living cells, bimolecular fluorescence complementation (BiFC) analysis was performed as described previously.^[^ ^47^ ^]^ The principle of BiFC analysis of STAT3‐STAT3 and STAT3‐SHF interactions was shown in a schematic view (Figure S3A, Supporting Information). U251 cells were transfected with STAT3‐nEGFP and STAT3‐cEGFP or SHF‐cEGFP for 16 h, fixed, stained with Hoechst 33 342 and DyLight 594 Phalloidin (F‐actin; CST), then visualized by a Leica TCS SP8 confocal microscope (40× objective). For control experiments, cells were transfected with STAT3‐ nEGFP and cEGFP‐empty vector for 16 h, fixed, and stained.

### Animal Experiments

Four week‐old female nude mice were purchased from the Shanghai Animal Center, Chinese Academy of Sciences, and maintained under specific pathogen‐free conditions. All mice were assigned randomly. For primary GBM tumor cells tumor tumorigenicity, the second or third passage of 3D‐cultured spheres were dissociated and counted. A total of 5 × 10^5^ cells was stereotactically injected into the brains of individual mice. Tumor formation was monitored by magnetic resonance imaging (MRI, Siemens) at 28 days after implantation. For U87 cell intracranial models, cells expressing the firefly luciferase gene were infected with SHF‐overexpressing lentivirus or control lentivirus. A total of 5 × 10^5^ cells were stereotactically implanted into the brains of individual mice. Tumor growth was monitored via bioluminescent imaging using IVIS Spectrum system and quantified by Living Image Software. To determine the effect of C16 on tumorigenicity, mice were injected i.p. once every 2 days with 10 µg of C16 or scrambled control (SC) from third day after tumor implantation. Tumor growth was monitored via bioluminescent imaging. Additionally, to examine the tumor inhibition of the SH2 construct, a total of 5 × 10^6^ U87 cells expressing ectopic SH2 or empty vector were injected s.c. into the flank of each nude mouse. Tumor growth was assessed by tumor diameters measured every 3 days. To measure tumor cell proliferation, mice were injected i.p. with EdU (10 mm, 0.4 mL) 24 h before sacrifice. At the end of experiments, mice were sacrificed via anesthetic overdose. Whole brains or tumors were removed, paraffin‐embedded, sectioned, and stained with hematoxylin and eosin (H&E) or EdU staining. Images were captured using a laser scanning confocal microscope. All animal care and handling procedures were performed in accordance with the National Institutes of Health's Guide for the Care and Use of Laboratory Animals. All animal experiments were approved by the Institutional Review Board of Nanjing Medical University (No.(2020)353). All animal experiments were performed by two technicians blinded to the treatment condition of the mice.

### Statistical Analysis

Data were expressed as mean ± SD. The difference between groups was performed using a one‐way analysis of variance (ANOVA) followed by a Newman Keuls’ multiple comparison test, the Kaplan–Meier method with log‐rank test, *χ*2‐test, or Spearman's rank correlation analysis. The F test was used to test variance equality. Differences were considered significant when *p* < 0.05. SPSS 16.0 package (IBM) and Graphpad prism 8.0 software (GraphPad Software) were used for all statistical analyses and data graphing, respectively.

## Conflict of Interest

The authors declare no conflict of interest.

## Ethics Statement

The study was approved by the Institutional Ethics Committee of Wuxi People's Hospital of Nanjing Medical University to the commencement of the study.

## Supporting information

Supporting Information

Supporting Information

Supplemental Table 1

Supplemental Table 2

Supplemental Table 3

Supplemental Table 4

Supplemental Table 5

Supplemental Table 6

Supplemental Table 7

Supplemental Table 8

Supplemental Table 9

Supplemental Table 10

## Data Availability

The data that support the findings of this study are available from the corresponding author upon reasonable request.
