# Supplementary material for: SHF Acts as a Novel Tumor Suppressor in Glioblastoma Multiforme by Disrupting STAT3 Dimerization
Source: Adv Sci (Weinh). 2022 Jul 17;9(26):2200169. doi: 10.1002/advs.202200169 (PMC9475553; doi:10.1002/advs.202200169)
Supplement: Supplementary file 1 — Supporting Information [file ADVS-9-2200169-s010.pdf]

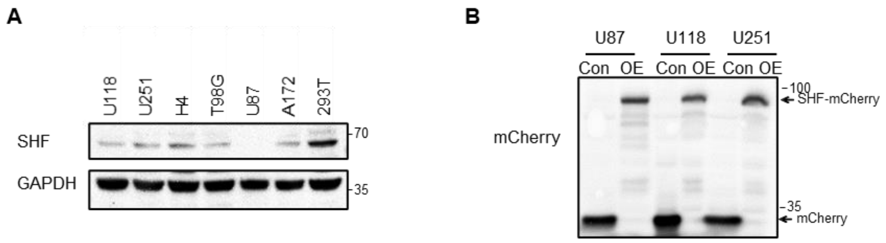

Supplementary Fig. 1 SHF expression in GBM cells lines and stable expressing cell lines.

A. Western blot assay of SHF expression in indicated GBM cell lines and 293T.

B. Western blot assay of ectopic SHF expression in stable expressing cell lines.

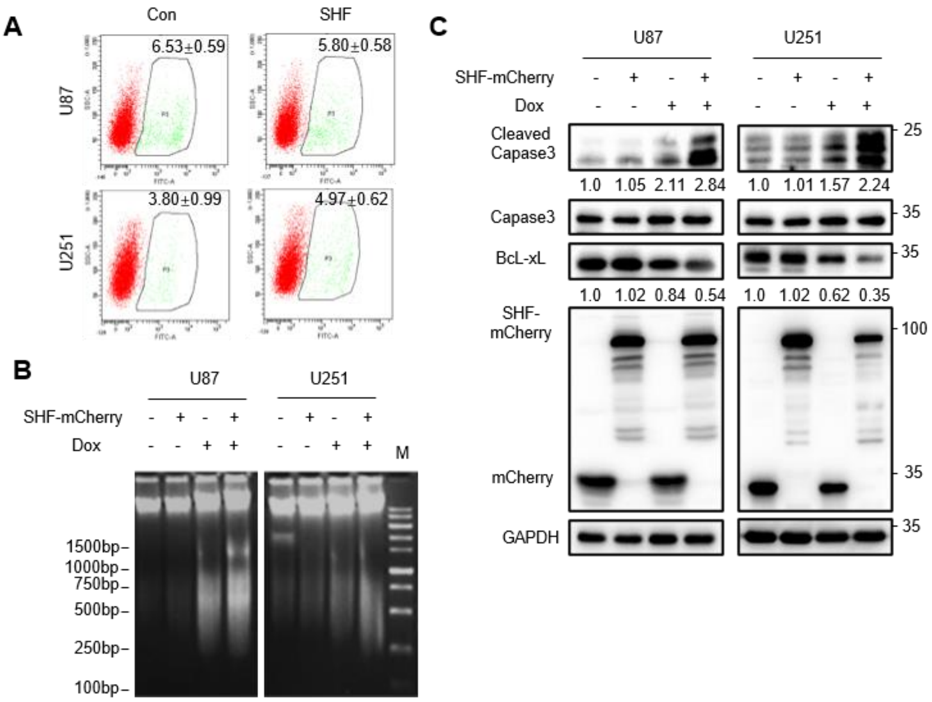

Supplementary Fig. 2 SHF expression enhances cellular apoptosis induced by DOX in GBM cells.

A. Flow cytometry assay of cells under normal culture conditions using Annexin V. The percentage of apoptosis was indicated (n=4).

B. Chromosomal DNA fragmentation analysis of cells treated with Dox (2 μM) for 48 h.

C. Western blot analysis of Caspaes-3, Cleaved Capase-3, and BcL-xL using the indicated antibodies. The relative quantification of the indicated proteins was listed.

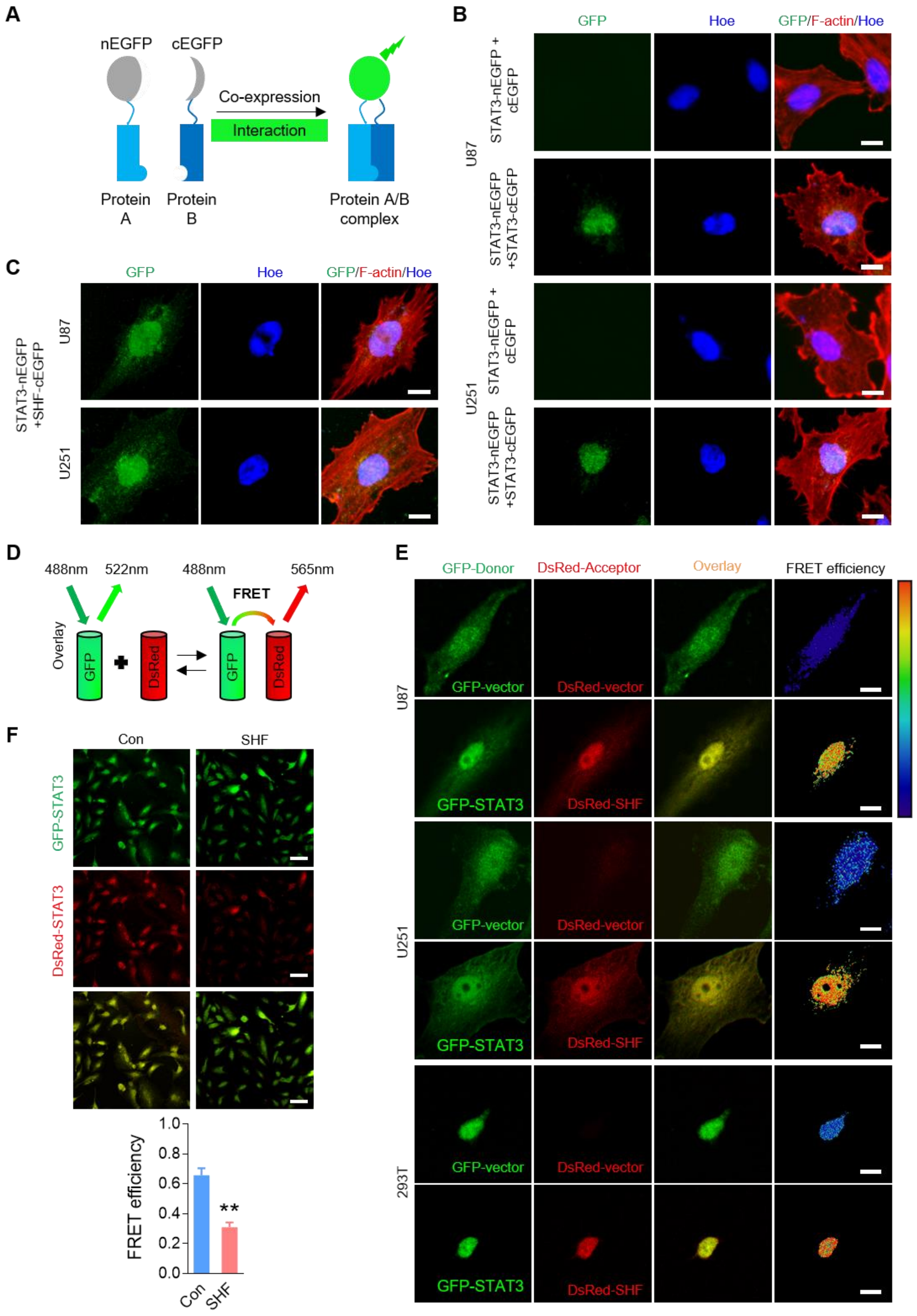

Supplementary Fig. 3 BiFC and FRET assay of the binding between SHF and STAT3.

A. Diagram indicating the BiFC assay for detection of SHF-STAT3 interaction.

B,C. Representative images of GBM cells transiently transfected with the indicated vectors for16 h. Bars, 10 um.

D. Diagram indicating the FRET assay for detection of SHF-STAT3 interaction.

E. Representative images of GBM cells and 293T cells transiently transfected with GFP-STAT3 and DsRed-SHF (or GFP-vector and DsRed-vector as negative control) for 24 h. Cells were excited at GFP-specific excitation wavelength (488 nm). The FRET ratios were calculated by reporting emission intensity of DsRed, measured at 565 nm, to the GFP emission peak measured at 522 nm. The pseudocolored ratio images (FRET images) represent the emission changes between negative control (GFP-vector and DsRed-vector) and STAT3-SHF interaction. The colorscale (top right) indicates that a shift toward the red end of the spectrum, which corresponds to higher FRET efficiency. Bars, 10 um.

F. Quantitative analysis of normalized FRET ratio in U251 stable cells transiently transfected with GFP-STAT3 and DsRed-STAT3 for 24 h (n=3, $**p < 0.01$). U251 cells expressing ectopic SHF showed a significantly lower 565/522 ratio compared with control cells. Bars, 50 um.

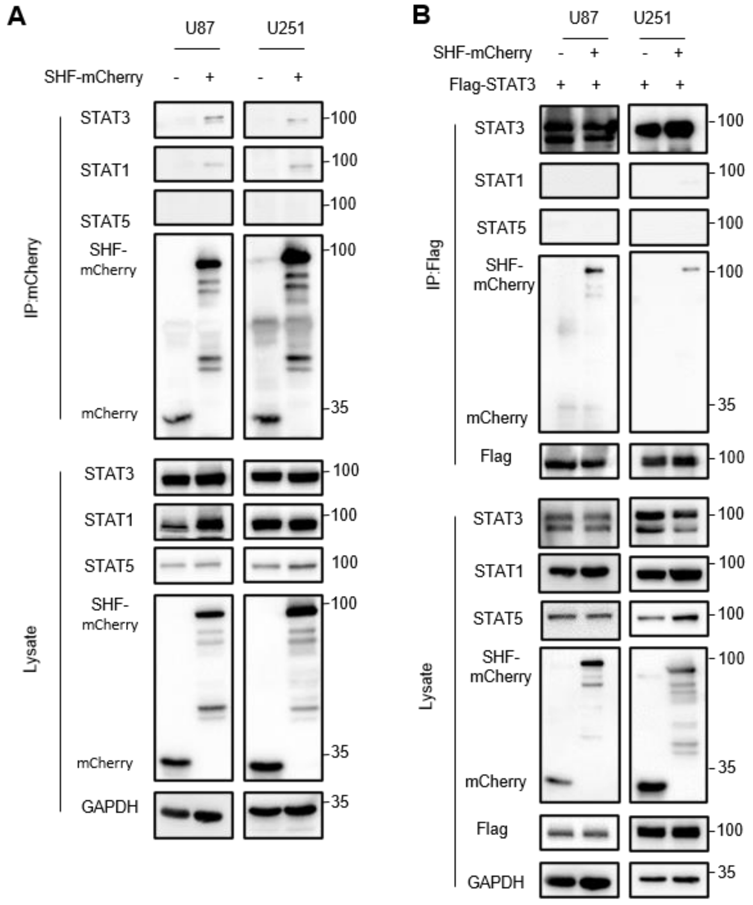

Supplementary Fig. 4 SHF has no effect on the interaction between STAT3 and STAT1 or STAT5.

A. IP analysis showing the interaction between SHF and STAT1/ STAT3 but not STAT5 in the indicated cells expressing ectopic SHF.

B. IP analysis indicating the effect of SHF on the interaction between STAT3 and STAT1 or STAT5. Indicated cells were analyzed by IP using STAT3 antibody.

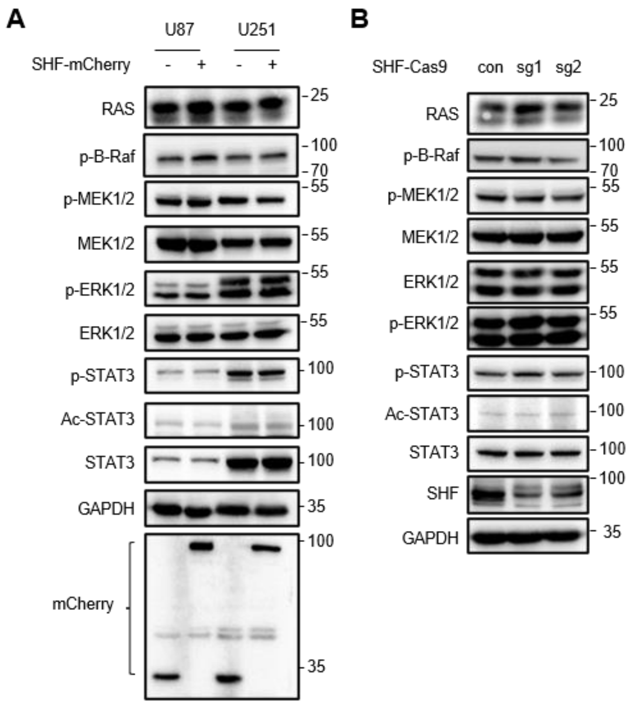

Supplementary Fig. 5 Western blot analysis of RAS/RAF/MEK signaling pathway in GBM cells expressing ectopic SHF (A) or with CRISPR/Cas9 mediated SHF knockdown (B) using the indicated antibodies.

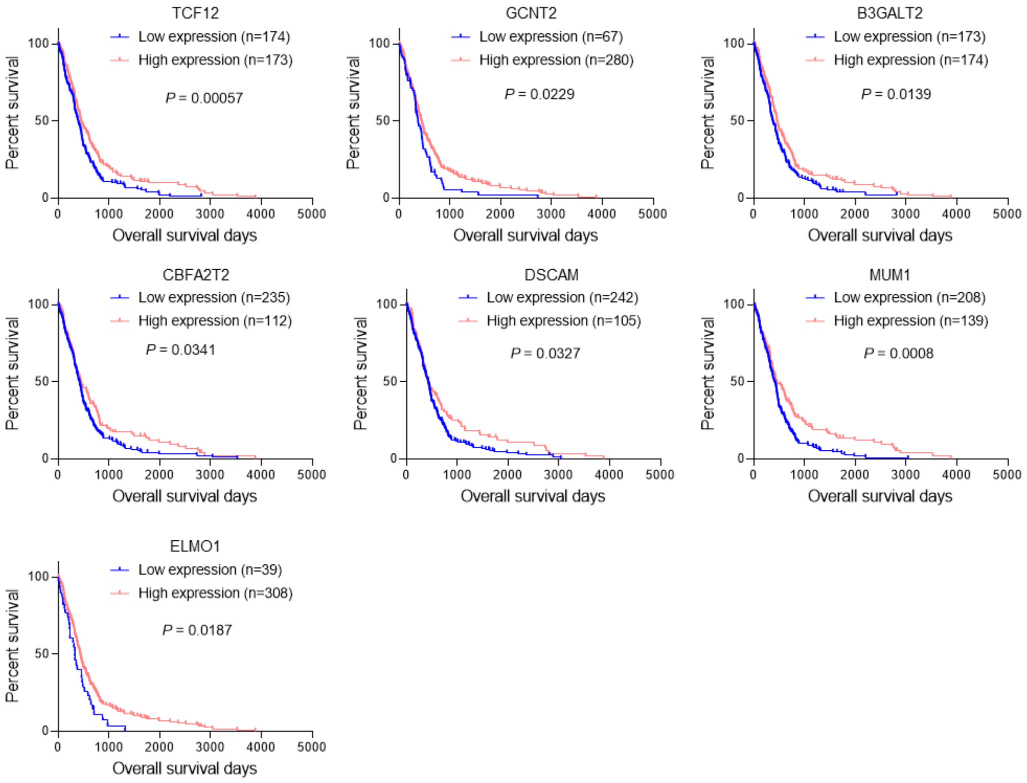

Supplementary Fig. 6 Overall survival analysis based on the indicated mRNA expression in the TCGA-GBM Affy Exon 1.0 ST dataset (Kaplan–Meier survival test).

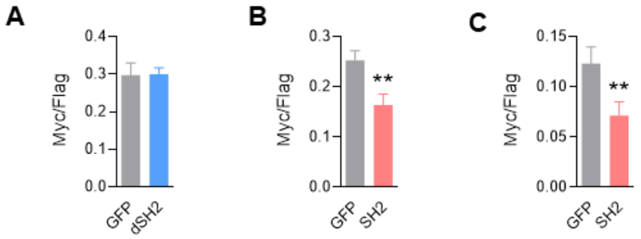

Supplementary Fig. 7 STAT3 dimerization and STAT3/DNMT interaction.

A. The effect of HA-dSH2 on the dimerization of STAT3 (n=3, Student's t test).

B. SH2 disrupts the dimerization of STAT3 (n=3, Student's t test, ***P* < 0.01).

C. SH2 disrupts the interaction between STAT3 and DNMT1 (n=3, Student's t test, ***P* < 0.01).

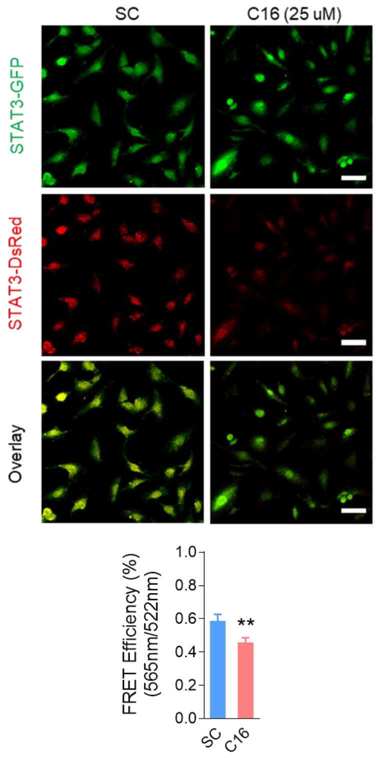

Supplementary Fig. 8 Quantitative analysis of normalized FRET ratio in U251 cells (n=3, ***P* < 0.01). U251 cells were transiently transfected with the indicated vectors for 24 h and treated with C16 for another 2 h. Bars, 50 um.

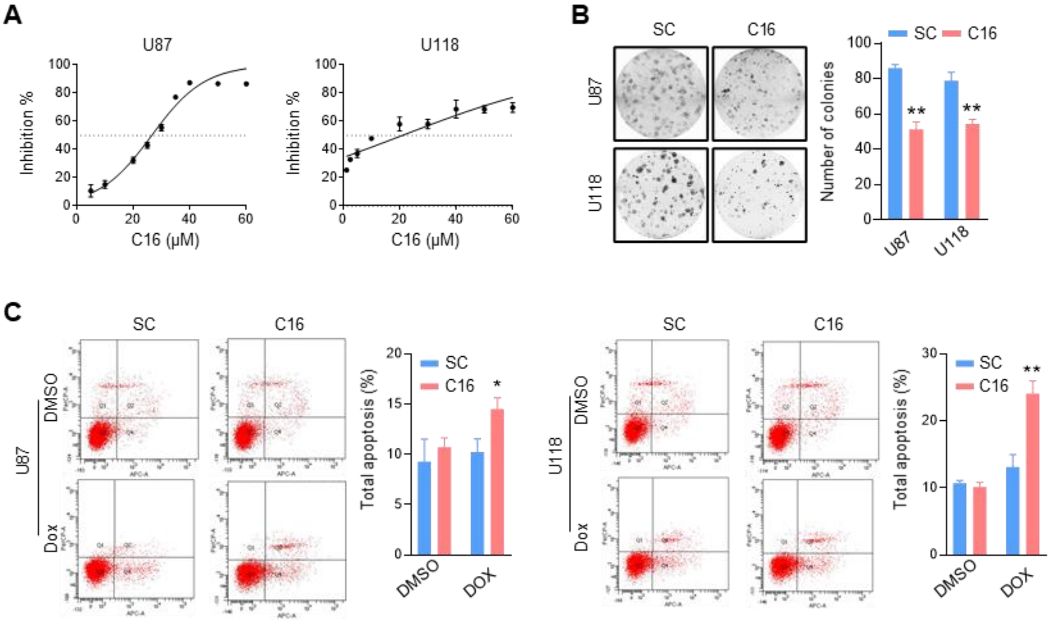

Supplementary Fig. 9 C16 inhibits tumor growth and promotes chemosensitivity *in vitro*.

A. C16 inhibited cell growth with concentration dependence in U87 and U118 cells (n=6).

B. Colony formation of cells treated with SC or C16 (left) and the accompanying statistical analysis (right; n=6, Student's *t*-test, ***P* < 0.01).

C. Annexin V staining flow cytometry assay of cells simultaneously treated with Dox with SC or C16 for 48 h (n=3, Student's *t*-test, **P* < 0.05, ***P* < 0.01).

Supplementary Table 1. Association between SHF expression and information of patients with GBM

Status		SHF expression[#], n		Total	*P* value*
		Low	High	n	
Gender	Male	14	15	29	0.793
	Female	15	14	29	
Age	<55 yrs	9	7	16	0.557
	≥55 yrs	20	22	42	
Survival[$]	≤1 yrs	11	10	21	0.462
	1-3 yrs	12	10	22	
	>3 yrs	4	8	12	

**P* values were analyzed by Chi-square test.

According to the immunoreactive scores (IRS) from IHC of GBM tissue array: the cutoff between LOW and HIGH was set at the median IRS of GBM tissues.

$ The total number of patients used in survival analysis was 55, those without survival information were excluded.

Supplementary Table 2. List of antibodies

Antigen	Primary Antibody	Dilution
SHF	Sigma; HPA046113; rabbit polyclonal	1:4000 for IHC/IF; 1:1000 for WB; 1:100 for IP
STAT3	Cell Signaling;9139; mouse monoclonal	1:1000 for WB; 1:100 for ChIP; 1:600 for IF
Phospho-STAT3 (Tyr705)	Cell Signaling; 9145; rabbit monoclonal	1:1000 for WB
Phospho-STAT3 (Ser727)	Cell Signaling; 94994; rabbit monoclonal	1:1000 for WB
Acetyl-STAT3 (Lys685)	Cell Signaling; 2523; rabbit monoclonal	1:1000 for WB
Histone H3	Cell Signaling; 4620; rabbit monoclonal	1:50 for ChIP
Normal Rabbit IgG	Cell Signaling; 2729	1:100 for IP or ChIP; 1:500 for IHC or IF
Normal Mouse IgG	ABCAM; ab188776	1:100 for IP; 1:500 for IF
GAPDH	ThermoFisher; MA5-15738-1MG; mouse monoclonal	1:2000 for WB
β-tubulin	Sigma; T5201; mouse monoclonal	1:200 for IF
Flag-tag	Abmart; M20008; mouse	1:5000 for WB; 1:200 for IP

	monoclonal	
GFP-tag	Abmart; M20004; mouse monoclonal	1:2000 for WB
mCherry-tag	GeneTex; GTX128508; rabbit polyclonal	1:2000 for WB; 1:50 for IP
HA-tag	Abcam; ab9110; rabbit monoclonal	1:5000 for WB; 1:50 for IP
His-tag	Proteintech; 66005; mouse monoclonal	1:5000 for WB
Myc-tag	Abmart; M20002; mouse monoclonal	1:5000 for WB; 1:500 for IP
GST	ABCAM; ab19256; rabbit polyclonal	1:2000 for WB
DNMT1	ABCAM; ab19256; mouse monoclonal	1:2000 for WB
DyLight 594 Phalloidin	Cell Signaling; 12877	1:20 for IF
KRAS	Proteintech; 12063; rabbit polyclonal	1:1000 for WB
MEK1/2	Proteintech; 11049; rabbit polyclonal	1:1000 for WB
phospho-MEK1/2 (Ser217/221)	Cell Signaling; 9154; rabbit polyclonal	1:1000 for WB
p44/42 MAPK (Erk1/2) (137F5)	Cell Signaling; 4695; rabbit monoclonal	1:1000 for WB
phospho-p44/42 MAPK (Erk1/2) (Thr202/Tyr204)	Cell Signaling; 4370; rabbit monoclonal	1:1000 for WB
Caspase 3	Cell Signaling; 9662; rabbit polyclonal	1:1000 for WB
Cleaved Caspase 3	Cell Signaling; 9664; rabbit polyclonal	1:1000 for WB
BcL-xL	ABCAM; ab178844; rabbit monoclonal	1:1000 for WB
STAT1	Cell Signaling; 9172; rabbit polyclonal	1:1000 for WB
STAT5	Cell Signaling; 94205; rabbit monoclonal	1:1000 for WB

Supplementary Table 3. Primers used for qPCR

Gene	Forward	Reverse
IL6	AGACAGCCACTCACCTCTTCAG	TTCTGCCAGTGCCTCTTTGCTG

JAK2	CCAGATGGAAACTGTTCGCTCAG	GAGGTTGGTACATCAGAAACACC
IKBKE	GGCTACAACGAGGAGCAGATTC	GGACGCTTGATACTTCTGCACG
GAPDH	CAACTTTGGTATCGTGGAAGGACTC	AGGGATGATGTTCTGGAGAGCC
TCF12	TCAGTGCGATGTTTTCCCCA	GGTTGACCACTTGTTCCCCA
DSCAM	CTCGGACTCAGGCAGCTATG	GGTCCTCAGTTCCTGTCACG
MUM	GCTGGAGAAAGAGTGCCAGT	GCTGGCCAGAAGGGGTATTT
CBFA2T2	AGGCAATGGAAAGTTGGTCC	GCACCATTCAGGGTAGGAGG

Supplementary Table 4. Primers used for qPCR of ChIP

Gene	Forward	Reverse
IL-6	ACACTTAGTGGAGGGCTTGG	AGCTTGCGTCTTGCTCCTAC
JAK2	GTCACAGCCGTTGTCTCCAC	GGCCTAGCGAATGTTTCTCCT
IKBKE	CCTCCAGCCTCCTAGGACAT	GCTCTTCAGAGACTGCTGGG

Supplementary Table 5. Primers used for qMSP

Gene	Forward	Reverse
TCF12 (methylated)	TAATAGAATTGATAAGTTGTAGGGAACG	CGAAAACGAAACTAATTATCGAA
TCF12 (unmethylated)	AATAGAATTGATAAGTTGTAGGGAAT	CAACCAAAAACAAAACTAATTATCAAAA
DSCAM (methylated)	GGTATTYGGYGTTTAGAATG	GCCCGCCTACTACCTAAATAC
DSCAM (unmethylated)	GTAGGTGGAGAGAGTTGTAGATGT	TACCCACCCACCTACTACCTAAATAC
MUM (methylated)	GTCGTAATAGCGGAGGATTC	AAACCGACGCTCCTTAAAAAC
MUM (unmethylated)	GGTTGTAATAGTGGAGGATTTG	CAACACTCCTTAAAAACACACACAT
