# Supplementary material for: SHF Acts as a Novel Tumor Suppressor in Glioblastoma Multiforme by Disrupting STAT3 Dimerization
Source: Adv Sci (Weinh). 2022 Jul 17;9(26):2200169. doi: 10.1002/advs.202200169 (PMC9475553; doi:10.1002/advs.202200169)
Supplement: Supplementary file 2 — Supporting Information [file ADVS-9-2200169-s004.pdf]

Figure 1J

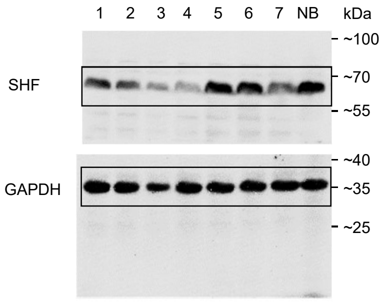

Figure 3G

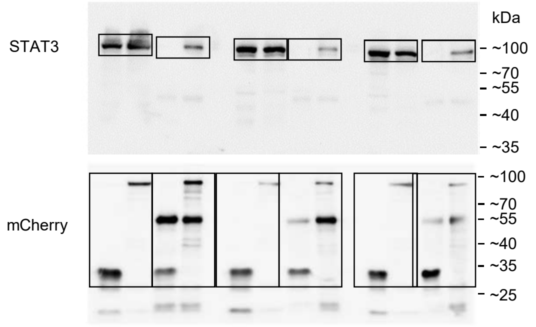

Figure 3F

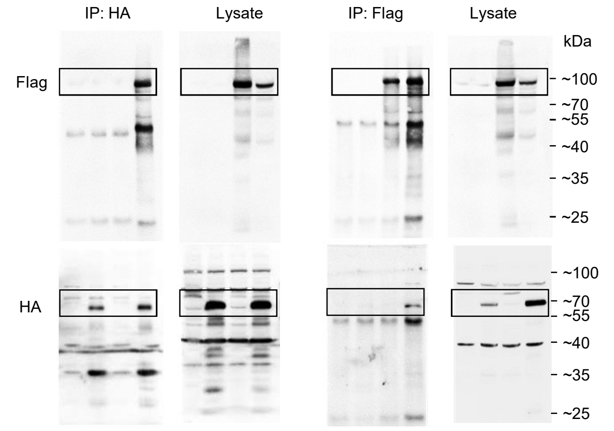

Figure 3J

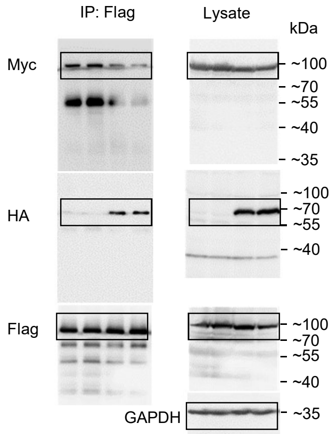

Figure 3I

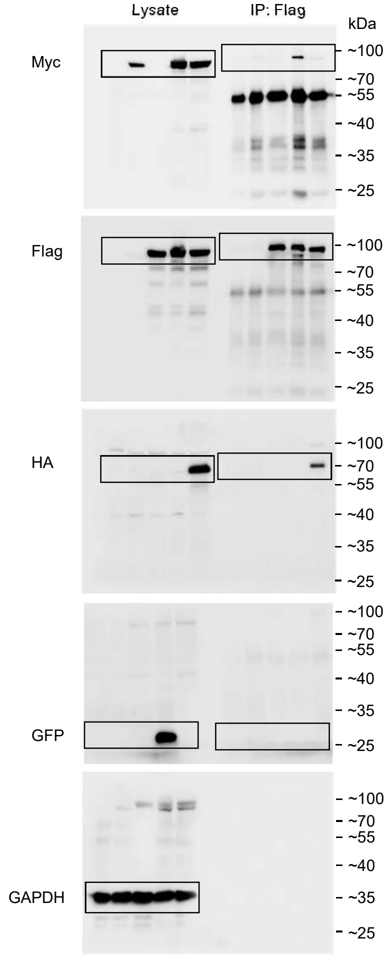

Figure 4A

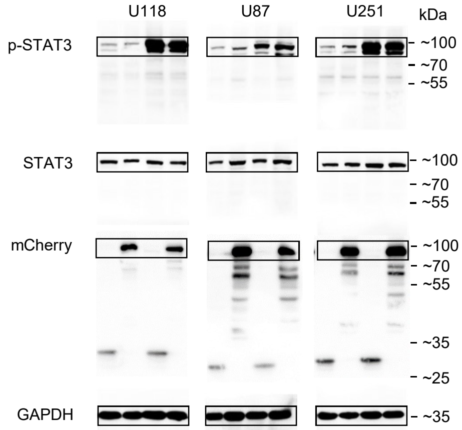

Figure 4B

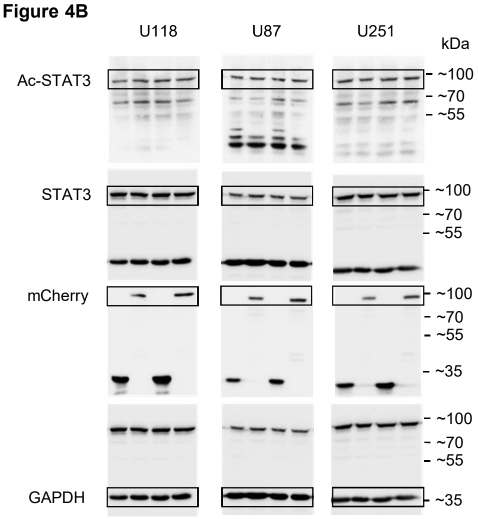

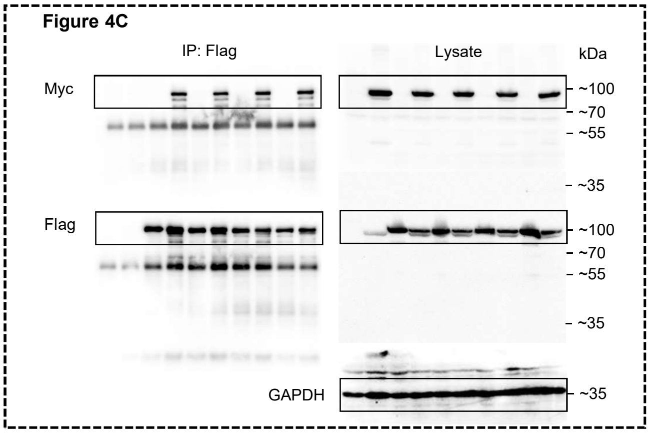
Figure 4C
IP: Flag
Lysate
kDa
Myc
Flag
GAPDH
~100
~70
~55
~35
~100
~70
~55
~35
~35

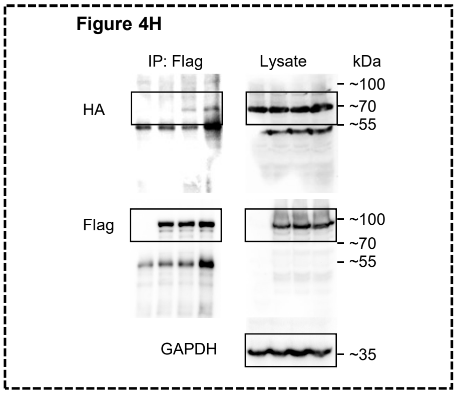
Figure 4H
IP: Flag
Lysate
kDa
HA
Flag
GAPDH
~100
~70
~55
~100
~70
~55
~35

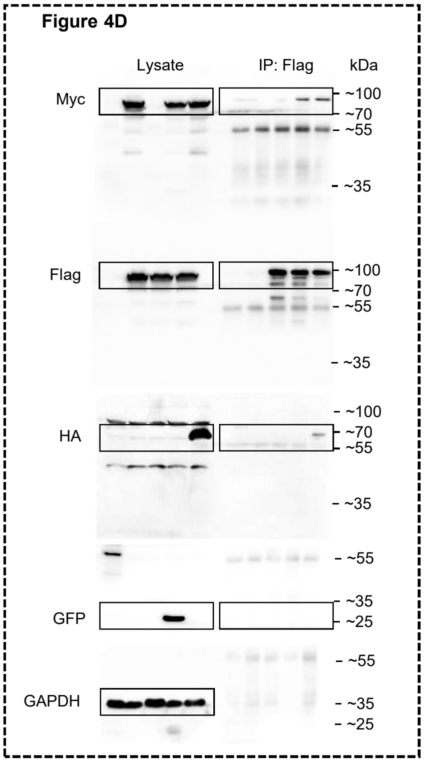
Figure 4D
Lysate
IP: Flag
kDa
Myc
Flag
HA
GFP
GAPDH
~100
~70
~55
~35
~100
~70
~55
~35
~100
~70
~55
~35
~55
~35
~25
~55
~35
~25

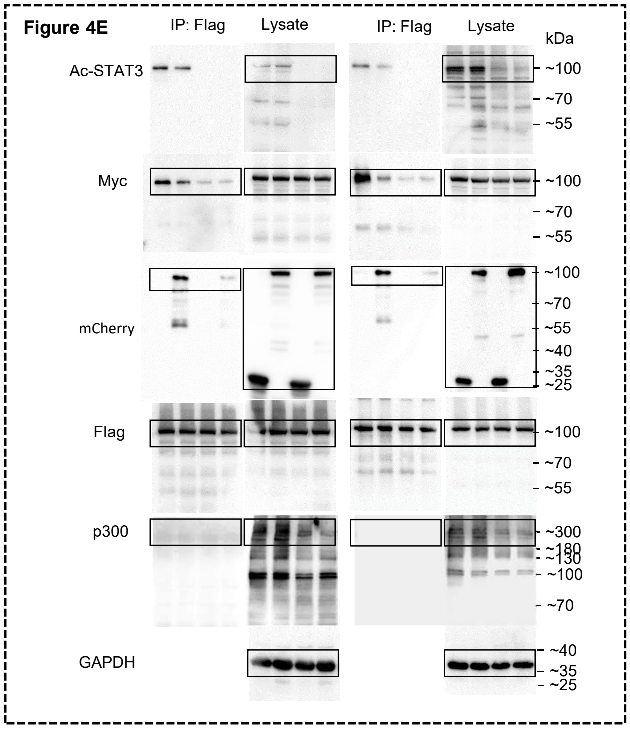
Figure 4E
IP: Flag
Lysate
IP: Flag
Lysate
kDa
Ac-STAT3
Myc
mCherry
Flag
p300
GAPDH
~100
~70
~55
~100
~70
~55
~100
~70
~55
~40
~35
~25
~100
~70
~55
~300
~180
~130
~100
~70
~40
~35
~25

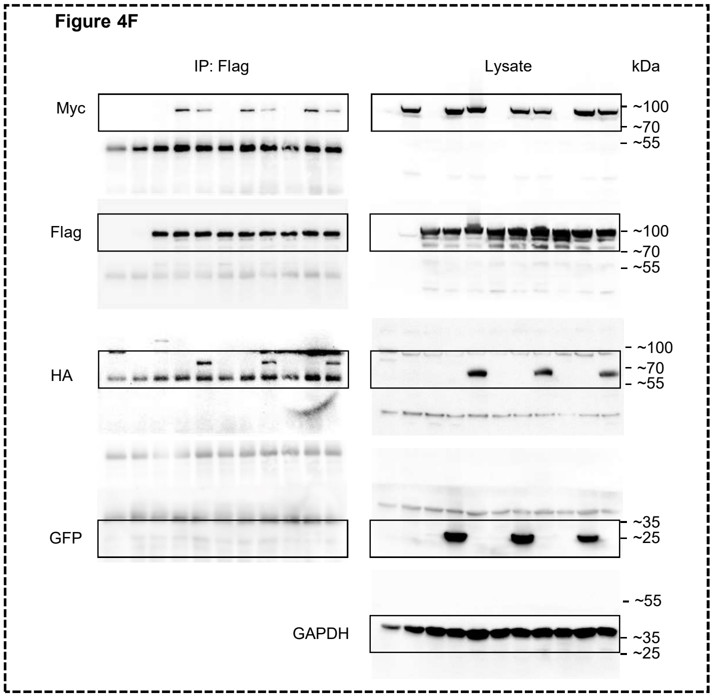
Figure 4F
IP: Flag
Lysate
kDa
Myc
Flag
HA
GFP
GAPDH
~100
~70
~55
~100
~70
~55
~100
~70
~55
~35
~25
~55
~35
~25

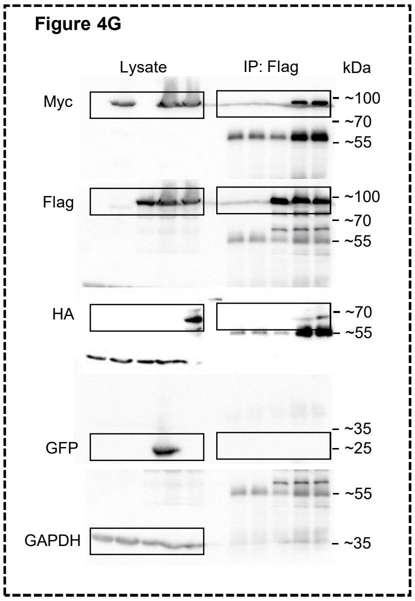
Figure 4G
Lysate
IP: Flag
kDa
Myc
Flag
HA
GFP
GAPDH
~100
~70
~55
~100
~70
~55
~70
~55
~35
~25
~55
~35

Figure 5A

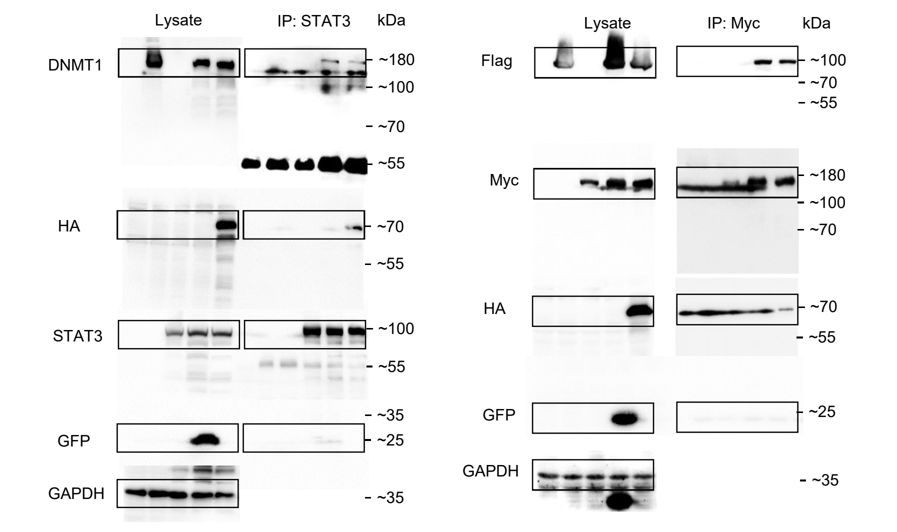

Figure 6B

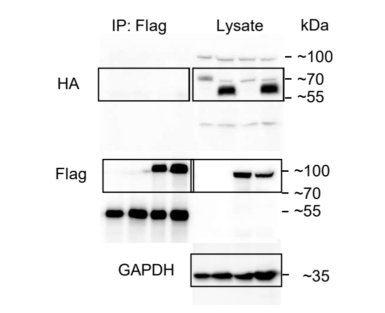

Figure 6C

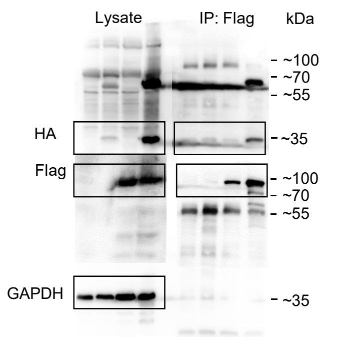

Figure 5B

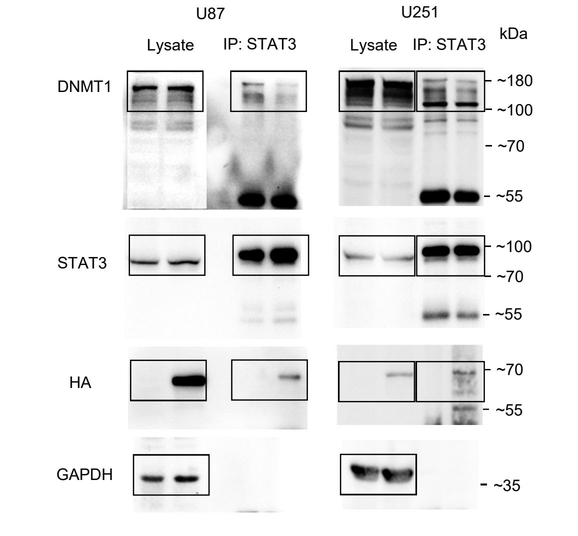

Figure 6D

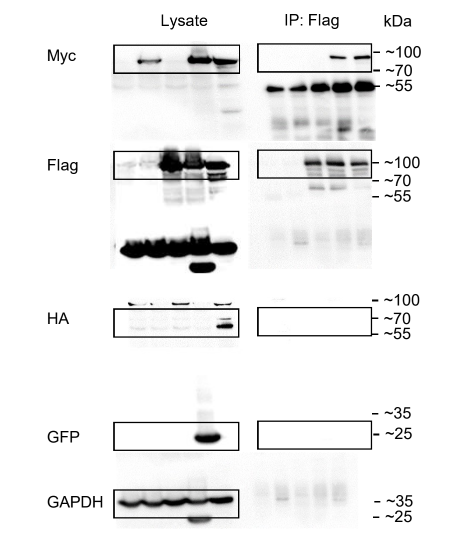

Figure 6E

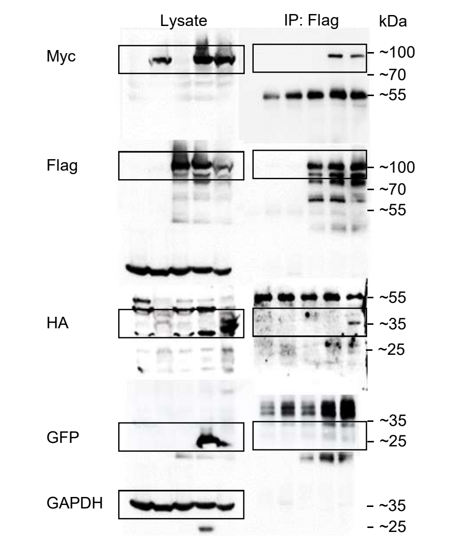

Figure 6G

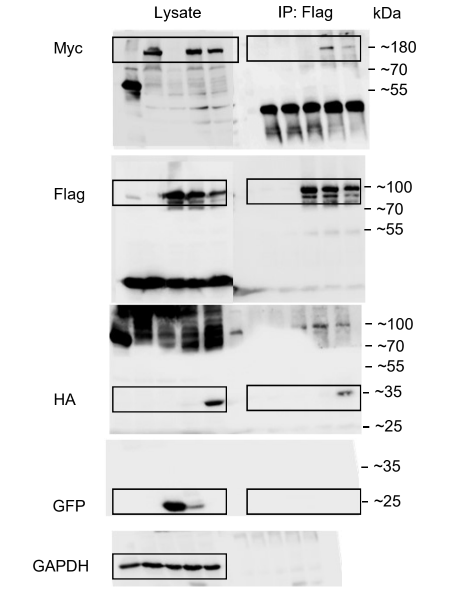

Figure 7E

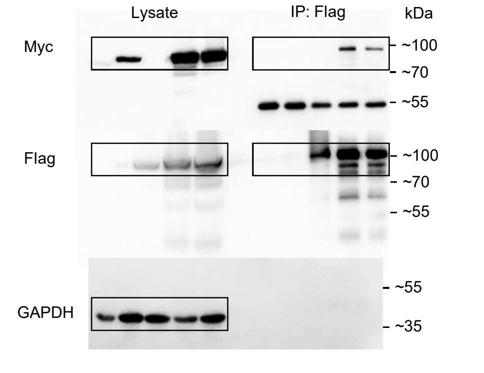

Figure 7G

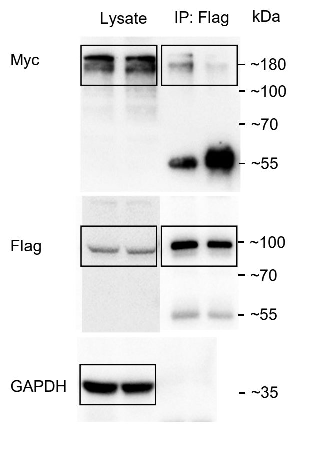

Figure 7F

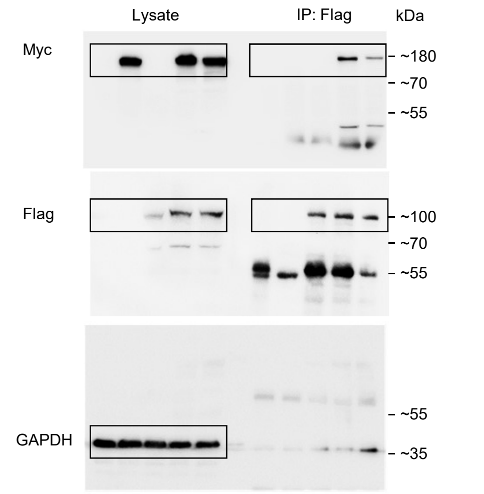

Figure 7D

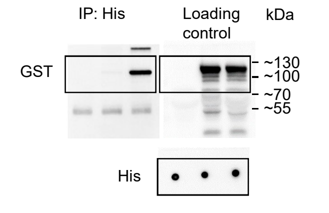

S Figure 1A

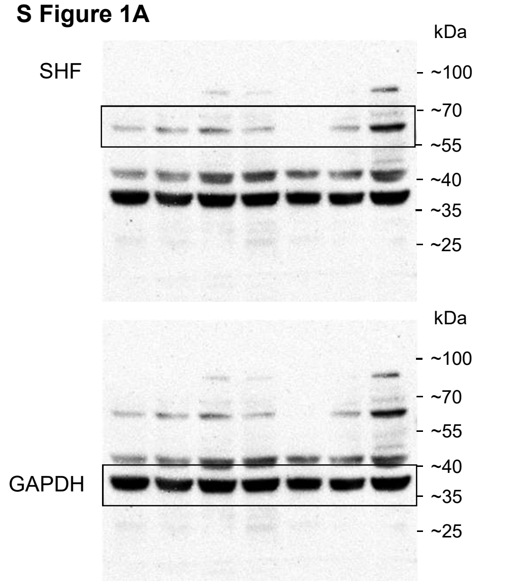

S Figure 1B

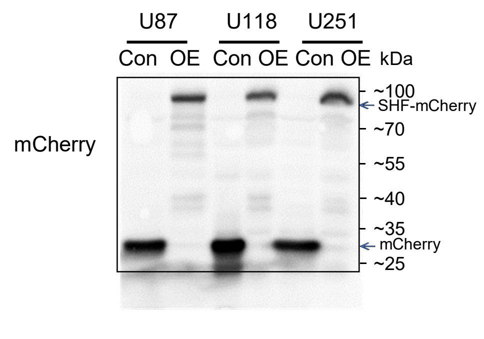

S Figure 2C

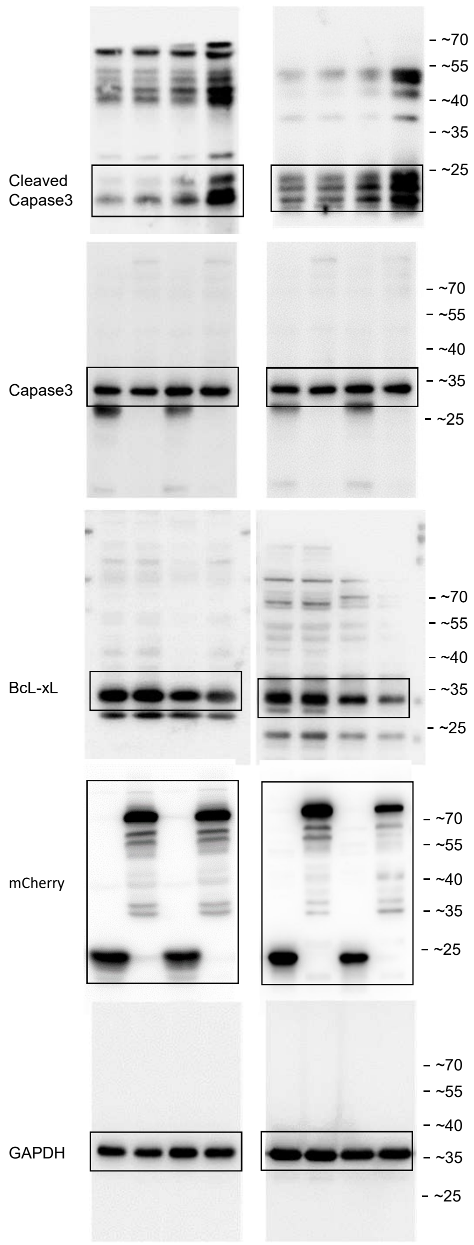

S Figure 4A

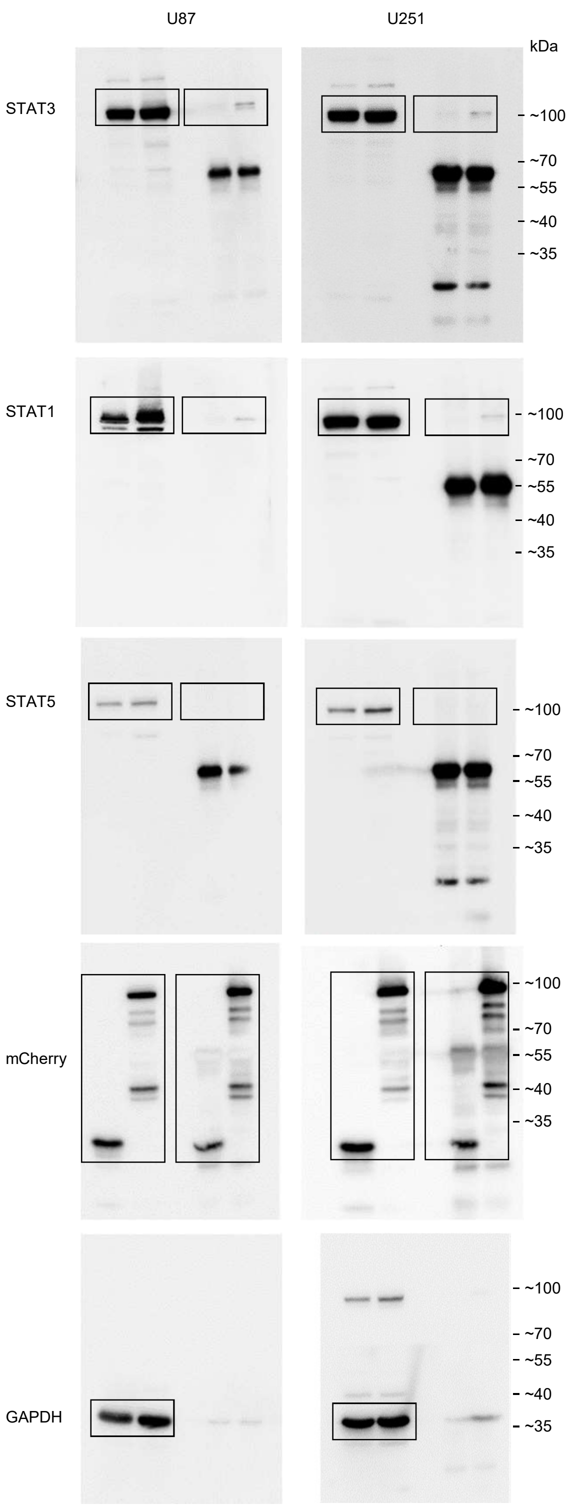

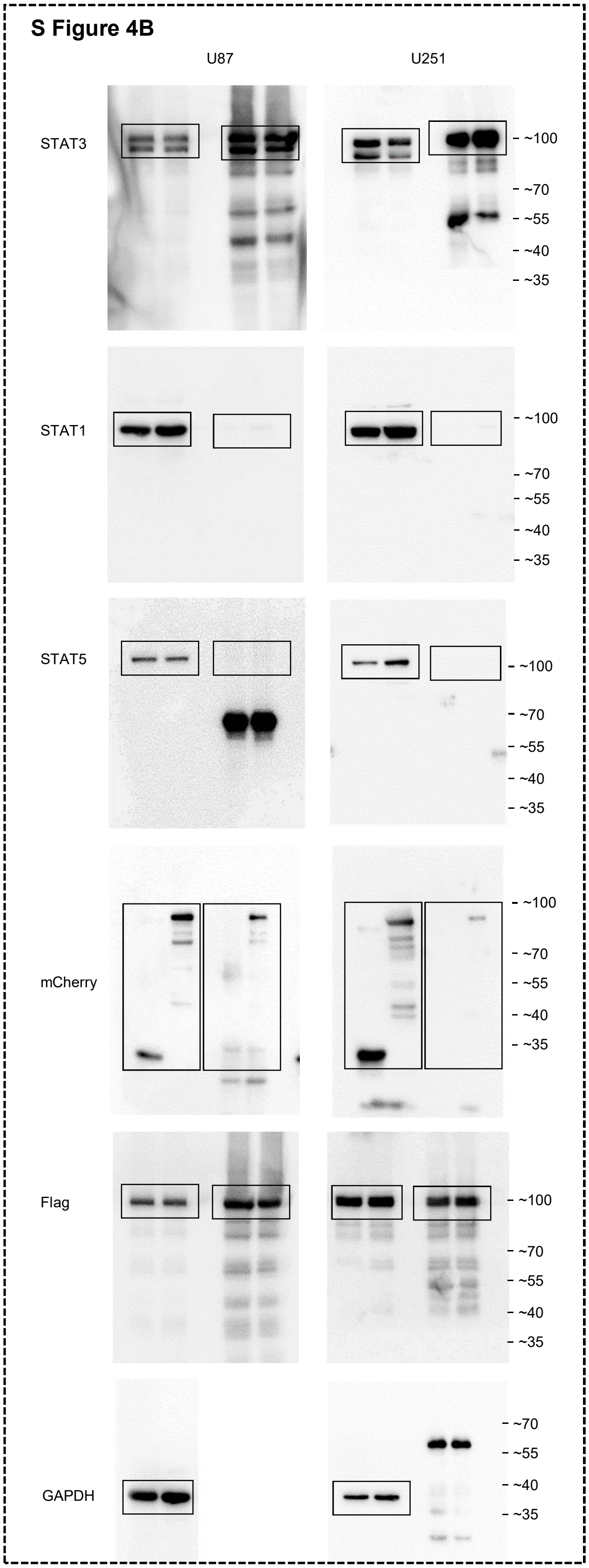
S Figure 4B
U87
U251
STAT3
STAT1
STAT5
mCherry
Flag
GAPDH
~100
~70
~55
~40
~35

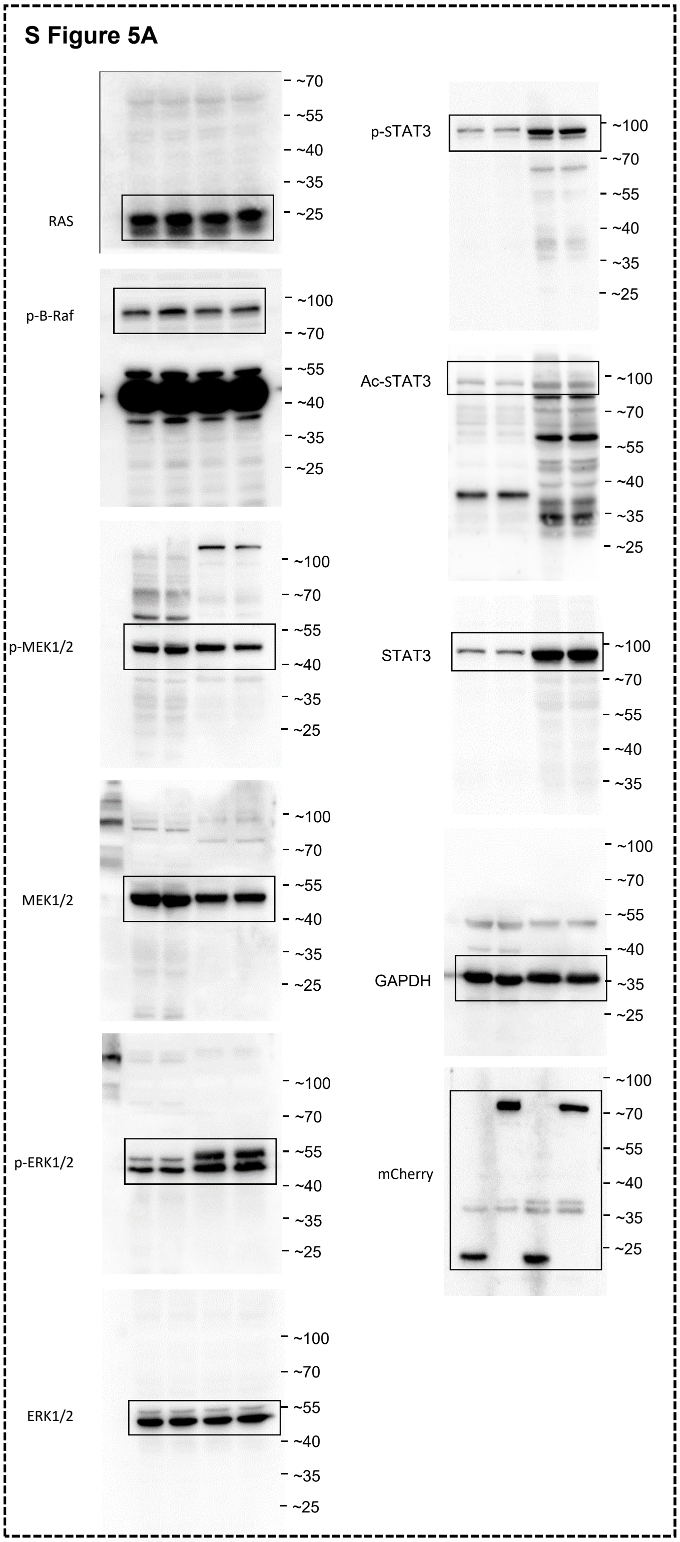
S Figure 5A
RAS
p-B-Raf
p-MEK1/2
MEK1/2
p-ERK1/2
ERK1/2
p-sTAT3
Ac-sTAT3
STAT3
GAPDH
mCherry
~100
~70
~55
~40
~35
~25

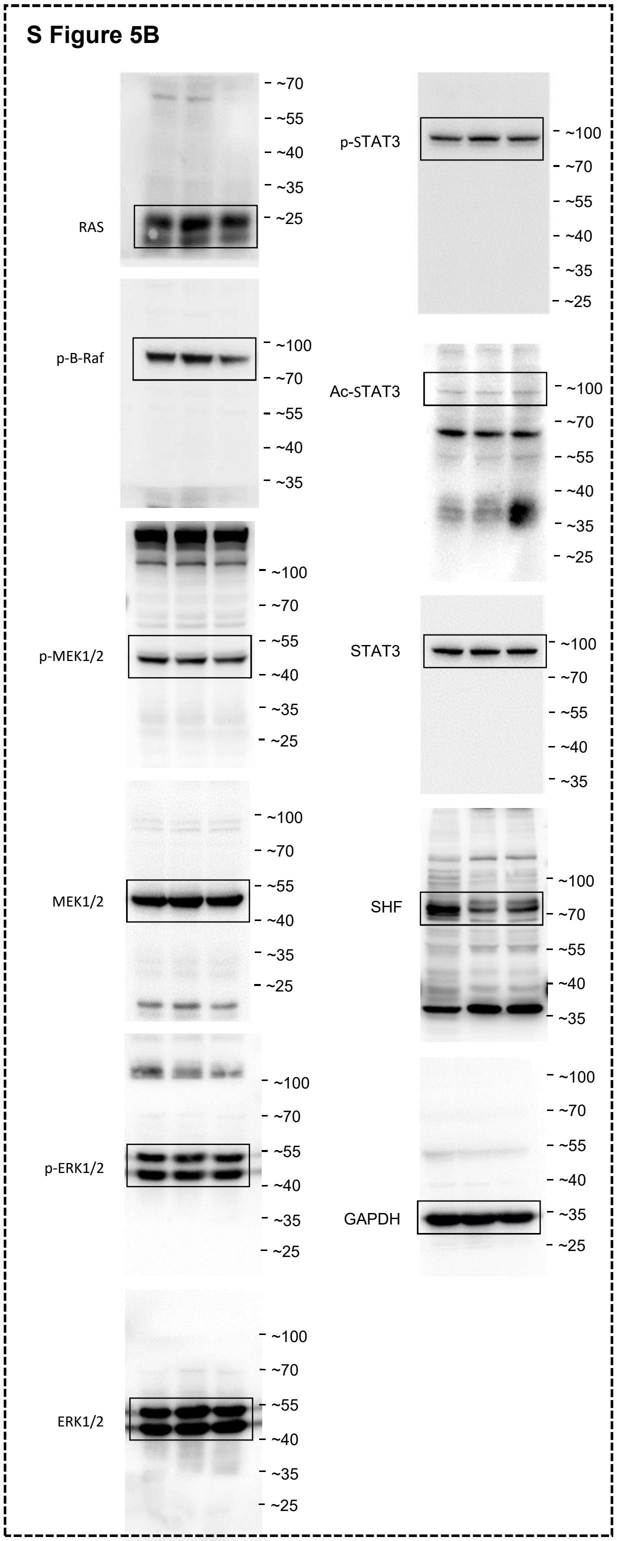
S Figure 5B
RAS
p-B-Raf
p-MEK1/2
MEK1/2
p-ERK1/2
ERK1/2
p-sTAT3
Ac-sTAT3
STAT3
SHF
GAPDH
~100
~70
~55
~40
~35
~25
