# Supplementary material for: SHF Acts as a Novel Tumor Suppressor in Glioblastoma Multiforme by Disrupting STAT3 Dimerization
Source: Adv Sci (Weinh). 2022 Jul 17;9(26):2200169. doi: 10.1002/advs.202200169 (PMC9475553; doi:10.1002/advs.202200169)
Supplement: Supplementary file 6 — Supplemental Table 4 [file ADVS-9-2200169-s012.pdf]

Supplementary Data 1. SHF expression and Clinical pathological information of GBM samples

No.	Overall survival (1=death, 0=alive)	Disease specific survival in days	Gender	Age	IRS of SHF
1	1	199	male	78	92.60
2	1	1172	male	37	20.21
3	1	2605	female	60	49.35
4	1	2360	female	58	49.75
5	1	2240	male	78	17.18
6	1	838	male	57	12.29
7	1	1295	female	61	81.91
8	1	838	female	61	46.15
9	1	2695	male	49	61.44
10	1	2240	female	66	66.02
11	1	1295	female	61	49.22
12	1	930	male	71	133.03
13	1	899	female	55	19.57
14	1	779	male	47	36.43
15	1	1022	male	50	37.46
16	1	382	female	47	24.65
17	1	48	female	67	35.53
18	0	756	female	56	56.69
19	1	229	male	63	24.98
20	0	330	female	64	46.73
21	0	288	female	69	37.48
22	0	104	female	32	22.35
23	0	76	male	58	38.96
24	0	72	male	66	71.46
25	0	61	female	56	36.17
26	1	3313	male	56	101.27
27	0	44	male	81	45.42
28	1	379	male	58	22.60
29	1	791	male	70	64.25
30	1	381	female	42	112.62
31	0	874	male	57	66.91
32	1	137	female	69	33.47
33	0	916	male	68	154.50
34	1	255	female	37	61.84
35	1	598	male	73	15.86
36	1	598	male	73	37.63

37	1	177	female	60	17.02
38	1	205	female	50	81.27
39	1	63	male	69	31.42
40	1	727	female	62	2.68
41	0	188	female	52	9.13
42	0	33	male	48	30.54
43	1	295	male	40	64.17
44	0	336	female	42	51.97
45	1	532	female	52	20.48
46	1	373	female	71	13.05
47	1	312	male	57	102.44
48	1	1629	male	78	24.04
49	1	838	male	59	16.18
50	1	687	male	60	40.86
51	1	657	male	60	59.25
52	1	425	male	54	31.85
53	1	2268	female	44	39.06
54	0	149	female	69	24.30
55	1	2360	female	58	17.91
56			female	71	107.83
57			female	71	29.54
58			male	58	5.64
